# Toward Generalizable Estimation of Behavioral Models with Parameter Dependencies

**DOI:** 10.1017/psy.2026.10089

**Published:** 2026-02-06

**Authors:** Stephen B. Broomell, Sabina J. Sloman, Lisheng He

**Affiliations:** 1 Psychological Sciences, Purdue University, USA; 2 Computer Science, University of Manchester, UK; 3 SILC Business School, Shanghai University, China

**Keywords:** behavioral modeling, context dependence of psychological theory, generalizability, parameter dependency, sampling distribution

## Abstract

Behavioral models are instrumental for studying human cognition, yet many inferences derived from such models fail to generalize. We argue that this is driven in part by the increasing complexity of behavioral models, where non-linearities and discontinuities create dynamic parameter interactions that limit the generalizability of inferences across different contexts, experiments, and datasets. We first demonstrate the problems that arise from parameter dependency. We then propose a new methodological framework for understanding the generalizability of behavioral modeling results using multivariate sampling distributions for the model parameters. We derive and validate novel sampling distributions for complex non-linear behavioral models by transforming the mimicry between different parameter values into the chances of one set of parameters being inferred from data generated by another set of parameters. Our approach is computationally scalable to evaluate how model estimates change across the parameter space and different experiments, which can limit the generalizability of experimental results. We then apply our approach to current behavioral models, revealing new theoretical insights. Using our approach, we reinterpret results from recent modeling work in decision-making and category learning. We conclude by discussing the implications of our proposed framework for building stronger, more generalizable psychological research and theory through behavioral modeling.

## 1 Introduction

Behavioral models play an instrumental role in the study of human cognition. Social and cognitive scientists use them to specify theories, make testable predictions (Navarro, 2021), and, by estimating model parameters, measure theoretical constructs (Busemeyer & Diederich, 2010). Beyond basic research, inferences drawn from behavioral models inform clinical interventions and public policy. The usefulness of model-based inferences lies in their generalizability, that is, the degree to which the resulting parameter estimates produce similar inferences and accurate predictions in different experimental contexts. Here, we define experimental contexts as the design features (i.e., the exact stimulus values and their distribution). Specifically, a model generalizes if we can make accurate predictions about how similar participants will react to new experimental stimuli.

Yet parameter estimates and inferences from behavioral models repeatedly fail to generalize. Examples span psychology and include models of clinical decision-making deficits (Humphries et al., 2015), perceptual discrimination (Miletić et al., 2017), the dynamics of multi-alternative, multi-attribute choice (Evans et al., 2019, 2020), temporal discounting (Vincent & Stewart, 2020), and decision-making under risk (Broomell & Bhatia, 2014; Krefeld-Schwalb et al., 2022; Walasek et al., 2021). In these examples, estimates of focal parameters depend on other non-focal parameters that are weakly constrained by the data, which leads to unstable or inaccurate estimates of both sets of parameters. In other scientific fields, such as systems biology and physics, research has characterized such parameters as “sloppy” (Gutenkunst et al., 2007; Machta et al., 2013). We argue that the increasing complexity of behavioral models has increased the “sloppiness” of parameters through unintended parameter dependencies (Krefeld-Schwalb et al., 2022). Applying such models to datasets without tools for fully understanding the degree to which the parameters can be separately identified leads to estimation biases and results that fail to generalize.

Advances in Bayesian model fitting have arguably opened the door to fitting parameters for complex, non-linear behavioral models that were difficult to fit using traditional approaches (Lee & Wagenmakers, 2014). The Bayesian approach provides a multivariate posterior distribution that represents the joint uncertainty of estimated parameters given the experimental data observed. This joint posterior distribution can contain dependencies between parameters, allowing researchers to express how changes in the values of non-focal parameters would affect their beliefs about a focal parameter. However, the Bayesian posterior distribution does not show how these dependencies might change if the experiment changed. This is because the Bayesian posterior distribution is conditional on the specific experimental stimuli and observed participant responses. Currently, there are no comprehensive frameworks for leveraging the posterior to account for dynamic changes in parameter dependency across contexts.

To facilitate generalizable estimation of behavioral models, we propose a general approach for accurate inference from complex behavioral models that clarifies the bounds of inferences drawn from one experiment when generalizing to another. To our knowledge, this is the first generally accessible approach for behavioral modeling that addresses limitations of current inference methods in the presence of parameter dependencies. While prior research has focused on the mean and variance of parameter estimates, we highlight the need to understand parameter dependencies as well as tools for estimating such dependencies and making generalizable inferences in their presence. Dependencies in parameter estimates arise from four different sources: variation in participant behavior (which is the object of interest to the modeler), the experimental stimuli, the model’s structure, and (for Bayesian modeling) the prior distribution. Two major implications of behavioral models that contain model structure dependencies are that (a) estimated parameters cannot be interpreted independently of the experimental stimuli, and (b) any individual parameter cannot be interpreted independently of the remaining statistically dependent parameters.

Historically, researchers have understood the behavior of estimated parameters using sampling distributions, which are the distributions of parameter values that could possibly be identified by fitting the model to a given dataset (Hogg & Craig, 1965; Lehmann & Casella, 1998). Sampling distributions capture dependencies due to the experimental stimuli and the model’s structure, clarifying how these contribute to the dependencies in estimated parameter values. The estimation properties of many broadly used parametric models (e.g., ANOVA and linear regression) are characterized by well-established asymptotic sampling distributions. Under certain assumptions, these distributions are treated as statistically independent in applied inference—for example, in the reporting of parameter-specific tests and confidence intervals in standard statistical software—so that much of the established statistical methodology treats sampling distributions for model parameters as independent.

Behavioral models that faithfully emulate non-linear, often non-differentiable and discontinuous cognitive processes will have statistically interdependent parameters that change non-linearly across the parameter space and interact in complex ways with experimental designs. This poses a methodological limitation for both Frequentist and Bayesian modeling approaches alike. This is because researchers currently cannot know (a) the expected behavior of the model prior to data collection (Broomell & Bhatia, 2014; Krefeld-Schwalb et al., 2022; Turner et al., 2013) and (b) how the model’s behavior will change when applied to different datasets (Broomell et al., 2019). The computational costs of simulating sampling distributions limit the investigation of the many distributions needed to reveal these behaviors, and computationally quick analytical sampling distributions for behavioral models are currently unavailable to behavioral researchers.

The fact that standard asymptotic sampling distributions are inadequate characterizations of estimator behavior in many practical scenarios is well recognized in several literatures, including Bayesian model selection (Myung et al., 2006), minimum description length (Grünwald, 2007), and work on misspecification and model selection (Claeskens & Hjort, 2003). For example, when the model is misspecified (i.e., the model cannot mimic the data-generating process), standard asymptotic sampling distributions incorrectly characterize the variance of estimator behavior (White, 1981). Our approach addresses a fundamentally different limitation of standard asymptotic sampling distributions due to experimental designs and their complex interaction with non-linear models. We, therefore, maintain the standard assumption of correct model specification. Within this setting, where the model is perfectly specified, it is still unclear how estimators will behave given scarce, noisy data that characterize most behavioral modeling settings.

To fill this gap, we develop and validate a novel analytical approach to generating sampling distributions based on finite observations using Gaussian models. We extend this analytical derivation to numerically generate sampling distributions for models that make binary predictions (e.g., models of choice, perception, or categorization), the latter of which covers a broad class of behavioral models with otherwise unknown sampling distributions. These novel sampling distributions enable researchers to quickly quantify the estimation accuracy of all model parameters, interpret parameter estimates in the presence of parameter interactions and dependencies, evaluate the effectiveness of a given experiment for counteracting the structural inter-parameter dependencies prior to data collection, and understand how model behavior will change when applied to different sets of stimuli.

We first introduce the statistical definition of parameter estimation and parameter sampling distributions. Second, we discuss how parameter dependencies create limitations for generalizing modeling results by dynamically altering the sampling distributions of parameters. Third, we present the derivation of our novel, analytically derived sampling distributions that can capture complex finite-sample parameter behavior and show how they relate to prior methods. We extend this approach to binomial distributions, where we use numerical methods to construct computationally quick and accurate sampling distributions. Fourth, we demonstrate how to analyze joint sampling distributions to make accurate inferences in the presence of parameter dependencies and clearly define the conditions under which they generalize. Fifth, we apply our approach and novel sampling distributions to prior work on modeling dichotomous behaviors, revealing new theoretical insights regarding the effect of experimental design on parameter dependency and recovery using the generalized context model (GCM) of category learning (Nosofsky, 1986) and the deviation of human behavior from the predictions of rational utility models using cumulative prospect theory (CPT; Tversky & Kahneman, 1992). Finally, we conclude by discussing the future directions for generalizable behavioral modeling and the limitations of models with dependencies.

## 2 Behavioral modeling

Let ***x*** be a matrix that represents the stimuli used to elicit response vector ***y*** from an experimental participant. Let a behavioral model be defined as a function *f* that maps the stimuli x and parameters θ to a predictive distribution over possible responses y such that *p*(***y**| **x***, ***θ***) = *f*(***y***; ***x***, ***θ***). When observed data $\left(x,y\right)$ are fixed and θ is unknown, this same function defines the likelihood $L\left(\theta |x,y\right)=f\left(y;x,\theta \right)$ .

### 2.1 Parameter estimation

The goal of parameter estimation is to find a vector of parameter values $\hat{\theta }$ in the theoretically possible set of values Θ such that the model predictions *p*(***y**| **x***, ***θ***) align closely with an observed response vector ***y***. An estimator is a specific function that defines the alignment between observed data and predictions that arise under each parameter in a model. In the Frequentist approach, researchers typically use an estimator for finding $\hat{\theta }$ that maximizes the likelihood function *L*( $\hat{\theta }$ | ***x***, ***y***) (Hogg & Craig, 1965). The maximum likelihood estimate (MLE) is a function of the observed responses ***y*** and the stimuli ***x***. The sampling distribution for this estimator, $p\left(\hat{\theta }|x,\theta_{0}\right)=\int_{R^{n}}p\left(\hat{\theta }|x,y\right)f\left(y;x,\theta_{0}\right)dy$ , characterizes the stochasticity in estimates of $\hat{\theta }$ that result from the randomness in samples of observed responses generated by a theoretically true set of parameters $\theta_{0}$ and set of stimuli x . The sampling distribution, therefore, only depends on the model *f*, the theorized true parameter $\theta_{0}$ , and the stimuli ***x***.

In the Bayesian approach, the researcher’s beliefs about ***θ*** are specified as a prior distribution, *p*(***θ***), which is updated based on observing ***y***. Instead of a point estimate, the researcher obtains an entire posterior distribution over the parameter space, *p*(***θ*** | ***x***, ***y***) $\propto L\left(\theta |x,y\right)$ *p*(***θ***) (Lee & Wagenmakers, 2014). The posterior distribution expresses the researcher’s degree of belief in a parameter ***θ*** given a model *f*, prior beliefs about ***θ***, stimuli ***x***, and observed responses ***y***. Unlike the sampling distribution, which averages across possible responses, the Bayesian posterior is constructed using a specific set of observed responses ***y***.

### 2.2 Estimation properties

Two important properties of an estimator are *bias* and *variance*, which are derived from the estimator’s sampling distribution. Estimation bias is defined as the expected difference between the value of the estimator and the true parameter value. This is formally defined as $E_{p\left(\hat{\theta }|x,\theta_{0}\right)}\left[\hat{\theta }-\theta_{0}\right]$ (Lehmann & Casella, 1998). The presence of estimation bias indicates that values of an estimated parameter from data are systematically smaller or larger than the true parameter value. Estimation variance is defined as the expected squared difference between the value of the estimator and the true parameter, formally defined as $E_{p\left(\hat{\theta }|x,\theta_{0}\right)}\left[\left(\hat{\theta }-E\left[\hat{\theta }|x,\theta_{0}\right]\right){\left(\hat{\theta }-E\left[\hat{\theta }|x,\theta_{0}\right]\right)}^{T}\right]$ (Lehmann & Casella, 1998). Estimation variance indicates the precision of parameter estimates: a larger variance indicates that the same experiment can result in a larger range of values of the estimator. Generally, researchers desire to minimize estimation bias and variance, either through the estimation method they employ or the experimental design used to estimate the parameters.

### 2.3 Parameter dependence

While the prior literature on experimental design has focused on minimizing estimation bias and variance (Chaloner & Verdinelli, 1995; Fedorov, 2010), the statistical dependence between estimators is under-investigated. Parameter dependencies are also critical in understanding model parameters’ behavior. The presence of statistical dependence complicates the interpretation of an estimator’s bias and variance: in the presence of statistical dependence, an estimator’s bias and variance depend on other parameter estimates. As reviewed above, statistical dependencies between estimates of behavioral model parameters are either a reflection of the natural distribution of parameters in a population or arise within the estimation process due to the experimental stimuli used to fit the model and/or structural dependencies within the model itself. In the context of a single experiment, all these sources can contribute to statistical dependence between the parameters estimated for a sample of research participants.

Dependencies that reflect intrinsic properties of the participant population will likely not cause any issues for generalizability if the research participants are sampled from the population of interest. However, dependencies that are driven by the experimental stimuli (values of stimuli ***x***) and/or structural properties of the model (predictive function *f*) can cause serious problems for model interpretation and generalizability. This is because even when sampling from the same population, the estimation bias and variance can change due to changes in the experimental setup. In the following subsections, we will show how these two sources of dependence limit generalizability from a participant sample to a participant population and (even within a participant sample) across contexts with different experimental stimuli. If not properly identified, these dependencies can be erroneously attributed to intrinsic properties of the participant population, leading to invalid inferences about the distribution of psychological constructs in a population. We will demonstrate how these two intertwined sources of dependence can be identified through visualization of the corresponding sampling distribution.

Because the sampling distribution $p\left(\hat{\theta }|x,\theta_{0}\right)$ depends only on the function *f*, the stimuli ***x***, and the true parameter $\theta_{0}$ , it isolates the effects of model structure and stimuli without being clouded by a specific observed response vector ***y*** or the researcher’s beliefs. Sampling distributions, therefore, provide valuable information about potential limits to model generalizability specifically arising from problematic sources of dependency. In contrast, the Bayesian posterior additionally reflects a specific set of responses and therefore includes dependencies in parameter values that might exist in the sample of participants that generated these responses.

#### 2.3.1 Stimulus-driven dependency

Stimulus-driven dependency is induced by the stimulus values presented to participants. The extent of the induced dependency will vary across experimental contexts.

Consider a basic linear regression model with mean-centered variables and no intercept, given by

(1) $$\begin{array}{c} y=b_{1}*x_{1}+b_{2}*x_{2}+\epsilon . \end{array}$$

**Figure 1:**
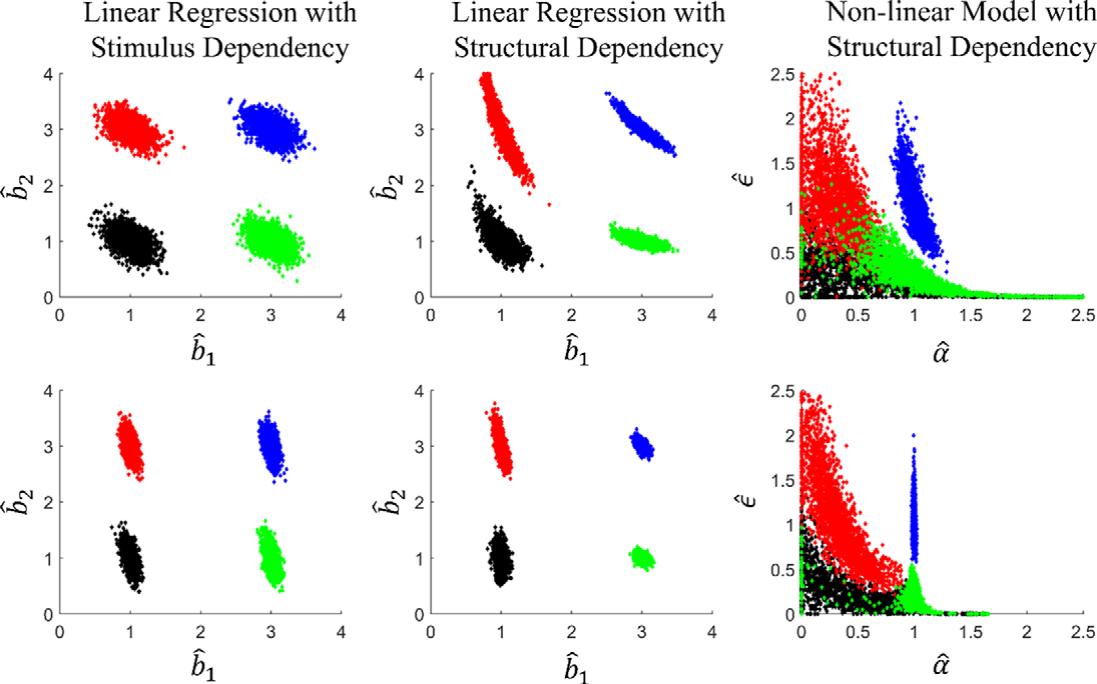
Sampling distributions from models containing parameter dependencies driven by different sources. Each column represents a different model. The top row shows baseline simulated parameter estimates. The bottom row shows simulated parameter estimates from the same modeling context, but with increased variance in the values of the variable labeled $x_{1}$ for the left/middle column and of payoff values for the right column. For $\left({\hat{b}}_{1},{\hat{b}}_{2}\right)$ , black = (1, 1), red = (1, 3), green = (3, 1), and blue = (3, 3). For $\left(\hat{\alpha },\hat{\epsilon }\right)$ , black = (0.3, 0.2), red = (0.3, 1), green = (1, 0.2), and blue = (1, 1). Figure 1 long description.

When ϵ is a Gaussian random variable, and all variables are independent of each other (i.e., cor( $x_{1}$ , $x_{2}$ ) = cor( $x_{1}$ , ϵ ) = cor( $x_{2}$ , ϵ ) = 0), the estimates of ${\hat{b}}_{1}$ and ${\hat{b}}_{2}$ are statistically independent. Dependency in the estimation of ${\hat{b}}_{1}$ and ${\hat{b}}_{2}$ is driven by any degree of correlation between the predictor variables $x_{1}$ and $x_{2}$ (i.e., the stimuli for this model). To illustrate this, Figure 1 (left column) displays simulated sampling distributions for ${\hat{b}}_{1}$ and ${\hat{b}}_{2}$ assuming the true data-generating process is given by parameter sets ( $b_{1}$ , $b_{2}$ ) of (1, 1), (1, 3), (3, 1), and (3, 3) with cor( $x_{1}$ , $x_{2}$ ) = 0.5 (plotted in black, red, green, and blue, respectively). The sampling distributions show parameter dependency and have identical properties (bias and variance) regardless of their location in the parameter space. The bottom half of Figure 1 displays simulated sampling distributions for the same model applied to the same sample of participants, but in which the variance of $x_{1}$ has been increased by a factor of 10. This change in experimental context reduces the estimation variance of ${\hat{b}}_{1}$ , but has almost no effect on the estimation properties of ${\hat{b}}_{2}$ .

In this example, the bias and covariance of the joint sampling distribution do not depend on the data-generating parameter value (the result would be similar for a Bayesian posterior). However, modeling results can suffer from inflated variance, and the interpretation of any single parameter depends on the values of other statistically dependent parameters. For example, fixing one of the parameters will shift the mean location of the other parameter (depending on where it is fixed) and alter the estimation variance.

#### 2.3.2 Model-driven dependency

Model-driven dependency is induced by the structure of the model itself. In its presence, an orthogonal design of stimuli does not necessarily eliminate the correlations in the sampling distribution. As the following examples will show, the structure of a model often interacts with the stimuli to produce dependencies. Model-driven dependency can persist across experimental contexts, and the extent and nature of the observed dependencies will vary as a function of the experimental stimuli. This means that certain experiments may be able to minimize their effect, but in some cases, this may not be possible.

We can add model structure dependency to the linear regression model from the previous example by altering Equation (1) so that both parameters interact to determine the effect of $x_{2}$ in the following way:

(2) $$\begin{array}{c} y=b_{1}*x_{1}+\left(b_{1}*b_{2}\right)*x_{2}+\epsilon . \end{array}$$

Both parameters $b_{1}$ and $b_{2}$ are directly linked to $x_{2}$ , creating a dependency between the values estimated for each parameter. If $x_{1}$ has no variance, then the model reduces to a form in which multiple $b_{1}$ and $b_{2}$ pairs generate the same probability distribution. In this case, parameter dependence creates the conditions for the experimental design to render the model unidentifiable. More generally, in the presence of model-driven dependence, the informativeness of the experimental design (e.g., the variance of $x_{1}$ ) determines whether parameters are merely estimated imprecisely or, in extreme cases, become unidentifiable.

Figure 1 (middle column) displays simulated sampling distributions for ${\hat{b}}_{1}$ and ${\hat{b}}_{2}$ assuming the same true generating parameters as before, but with cor( $x_{1}$ , $x_{2}$ ) = 0. More extreme values of $x_{1}$ provide more information about the unique effect of $b_{1}$ , which in turn provides more information about the unique effect of $b_{2}$ . This is not the case with the regression model in Equation (1). In this example with model-driven dependency, the estimation properties are dynamic across the parameter space (comparing the four distributions within either middle panel) and the stimulus space (comparing the middle top to the middle bottom panel). In this linear example, although the estimators’ variance and correlation change across the parameter and stimulus spaces, the mean estimates remain unbiased (i.e., are roughly equal to the true parameter values), and so would reasonably generalize across experimental contexts.

#### 2.3.3 Model-driven dependencies in behavioral models

The estimators’ properties can become even more unstable for non-linear models, which are characterized by more convoluted model-driven dependencies (Seber & Wild, 1989). Consider a simplified behavioral model that predicts choices between two lotteries defined by a set of outcomes and their associated probabilities of occurrence (e.g., a 0.8 chance of winning $4 and $0 otherwise), summarized by the vector 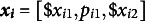. Each outcome in the lottery is first transformed into a subjective value by a power function (controlled by the free parameter a ):

(3) vxij|α=\lbrace\kern0.84em_^xija\kern0.72emif\;_xij≥0−−xija\kern0.36emif\;_xij<0.

Then, the subjective value of the lottery is computed as the expected subjective value of the outcomes given by

(4) $$\begin{array}{c} V\left(x_{i}|\alpha \right)=v\left(x_{i1}|\alpha \right)*p_{i1}+v\left(x_{i2}|\alpha \right)*\left(1-p_{i1}\right). \end{array}$$

Finally, the probability of choosing the first lottery is defined by the logistic function (controlled by the free parameter ϵ ) as

(5) $$\begin{array}{c} p\left(x_{1}\;prefered\backslash kern0.17emto\;x_{2}|\alpha ,\epsilon \right)=\frac{e^{\epsilon *V\left(x_{1}|\alpha \right)}}{e^{\epsilon *V\left(x_{1}|\alpha \right)}+e^{\epsilon *V\left(x_{2}|\alpha \right)}}. \end{array}$$

This model has two free parameters, α and ϵ , each of which controls a non-linear function. Further, there is a structural dependency such that the logistic function is applied to the lottery that has been transformed by the power function. Therefore, tuning the parameter ϵ depends on the output of the function controlled by the parameter α. Figure 1 (right column) displays simulated sampling distributions for $\hat{\alpha }$ and $\hat{\epsilon }$ assuming the true generating process is given by parameter sets ( α , ϵ ) of (0.3, 0.2), (0.3, 1), (1, 0.2), and (1, 1) plotted in black, red, green, and blue, respectively. Across both the stimulus and parameter space, the sampling distributions from this non-linear, structurally dependent behavioral model produce parameter estimates whose mean, variance, and correlation changes are orders of magnitude larger than changes that would arise from natural variation in a participant population. As an example, when α = 0.3, the mean estimates for $\hat{\alpha }$ increase from 0.30 (unbiased) to 0.59 (biased) when ϵ changes from 1.0 to 0.2. This bias implies that the estimated model parameter represents much more risk-seeking behavior than the true parameter value underlying the data-generating process. While this result replicates that of Krefeld-Schwalb et al. (2022), showing this type of distorted parameter estimation for similar models, we also find that structural dependency leads to changes in the magnitude of this distortion across both the stimulus and parameter space. This type of structural dependence can be seen in many behavioral models that transform stimuli to subjective values and transform subjective values into choice probability (such models are discussed in detail in Section 5 and in Krefeld-Schwalb et al., 2022).

The presence of structure-driven dependency can, therefore, place very strong limits on the generalizability of non-linear models, even in circumstances where the model is correctly specified (Seber & Wild, 1989). There are currently no approaches within the psychological literature for identifying or addressing this problem. Exactly how dependence affects model generalizability will be different for every model. For example, estimator bias and variance remain more stable with the structurally dependent linear model compared to the structurally dependent non-linear model. Therefore, modelers need to assess how the properties (bias, variance, and correlation) of parameter estimates from any structurally dependent model will change across experimental contexts. We propose a novel method for estimating sampling distributions, which is based on finite-sample properties of the estimators and so reflects both stimulus- and model-driven dependencies, which would be more difficult to isolate with existing methods like Bayesian posteriors.

## 3 A new approach for estimating sampling distributions

As shown in the previous section, parameter dependencies due to the stimuli and the model can be isolated from other sources and observed using sampling distributions. In the presence of structural dependencies, researchers will need to investigate several sampling distributions to understand how the modeling context may limit generalizability. A brute force approach to constructing a sampling distribution $p\left(\hat{\theta }|x,\theta_{0}\right)$ is to simulate sufficiently many sets of responses from the model under $\theta_{0}$ and estimate $\hat{\theta }$ for each response set. However, simulations require time to generate an accurate representation of the distribution. Therefore, we derive and validate a novel approach to generating computationally efficient sampling distributions for non-linear and discontinuous behavioral models with binomial likelihoods, a common type of behavioral model that suffers from dependencies (Krefeld-Schwalb et al., 2022).

Researchers rely on Fisher information (FI) to understand estimation properties (Li et al., 1996; Veldkamp & van der Linden, 2002). The FI function (Lehmann & Casella, 1998) defines the asymptotic sampling distribution as $\hat{\theta }$ *~ N* $\left(\theta_{0},{\left[N*FI\left(\theta_{0}\right)\right]}^{-1}\right)$ (Gelman et al., 1995). FI leverages the behavior of the likelihood function at $\theta_{0}$ to approximate the sampling distribution across the entire parameter space. The accuracy of the obtained sampling distribution is only guaranteed in the limit of infinite data. However, behavioral scientists must make inferences based on limited samples, so the assumption of many observations is often not valid, and even under correct model specification, it can lead to sampling distributions that are poor approximations. We, therefore, introduce a new method to construct valid sampling distributions that better reflect the limited number of observations in psychological studies.

FI can be expressed as the local curvature (second derivative) of the Kullback–Leibler (KL) divergence (Cover & Thomas, 1991; Kullback & Leibler, 1951) of the likelihood function at $\theta_{0}$ . Following the approach of Broomell and Bhatia (2014), we base our finite-sample approximation of the sampling distribution on the KL divergence between parameter values both locally and globally. The KL divergence from a distribution p to a distribution q is written $D_{KL}\left(p\|q\right)$ . Divergence between identical distributions is zero and increases with differences between the distributions. We compute the KL divergence between the likelihood of a focal parameter, $\theta_{0}$ , and the likelihood of another parameter value in Θ , which we denote by $\theta_{1}$ , as

(6) $$\begin{array}{c} D_{KL}\left(L\left(\theta_{0}|x,y\right)\backslash VertL(\theta_{1}|x,y\right))=E_{p\left(y|\theta_{0},x\right)}\left[\ln\left(\frac{p\left(y|\theta_{0},x\right)}{p\left(y|\theta_{1},x\right)}\right)\;\right]\;for\;\theta_{0},\theta_{1}\in \Theta , \end{array}$$

where the expectation is taken across potential observed responses ***y***. By varying $\theta_{1},$ Equation (6) results in a surface that covers the parameter values of theoretical interest in Θ . We convert this surface into a sampling distribution under which the probability of estimating $\theta_{1}$ when the true parameter is $\theta_{0}$ is

(7) $$\begin{array}{c} p_{KL}\left(\hat{\theta }=\theta_{1}|x,\theta_{0}\right)\propto \backslash exp\left[-\alpha *D_{KL}\left(L\left(\theta_{0}|x,y\right)\|L\left(\theta_{1}|x,y\right)\right)-\beta \right], \end{array}$$

where α and β represent corrections needed to transform the KL divergence into what we call a KL sampling distribution. Equation (7) mirrors results from large deviations theory (Dembo & Zeitouni, 1989) linking the probability that data generated by one distribution may be mistaken for data from another to the exponentiated KL divergence between the two distributions. A detailed exposition of our approach and the analytic derivation of the correction terms are provided in the Supplementary Material. To the best of our knowledge, this is the first approach to constructing a sampling distribution that requires neither asymptotic behavior nor computationally expensive simulations. Since these KL sampling distributions do not need to be simulated, they can be leveraged in other simulation analyses, facilitating deeper investigation of behavioral models.

Like asymptotic FI sampling distributions, KL sampling distributions approximate finite-sample behavior, but they do so more accurately. FI is estimated by the instantaneous curvature of the KL divergence surface defined in Equation (6) at the point $\theta_{0}$ and its inverse approximates the covariance structure of the asymptotic sampling distribution (Gelman et al., 1995). In non-linear models, accurately capturing the global features of the sampling distribution requires incorporating more fine-grained curvature of the KL divergence surface, even under correct model specification (Seber & Wild, 1989). Our approach leverages the full KL divergence surface, as opposed to only the instantaneous curvature, thereby capturing non-local geometric features that elude the standard FI approach.

**Figure 2:**
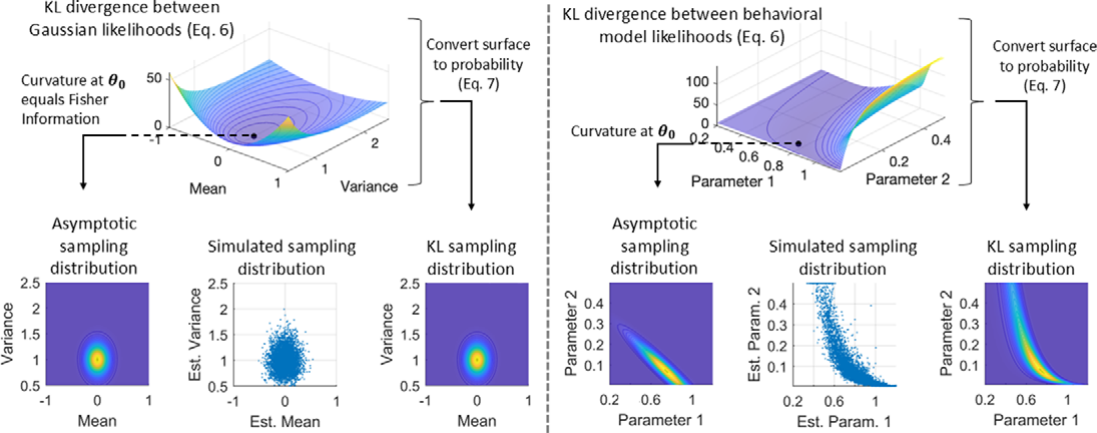
Depiction of the KL divergence surface defined in Equation (6) above the FI and KL sampling distributions derived from this surface along with an empirically simulated sampling distribution. Lighter colors indicate higher points on the surface. (Left) Gaussian model where both the asymptotic distribution and KL sampling distribution are equivalent approximations of the true sampling distribution. (Right) Non-linear behavioral model where the asymptotic distribution is an inaccurate approximation of the true sampling distribution, but the KL sampling distribution is a close approximation of the true sampling distribution. Figure 2 long description.

The connection between the KL and FI sampling distributions is displayed in Figure 2. The Gaussian model on the left can be perfectly summarized by both asymptotic FI and our KL sampling distribution. The behavioral model (Equations (3)–(5)) on the right is non-linear, reducing the validity of FI because the instantaneous curvature of the KL divergence surface at $\theta_{0}$ cannot accurately summarize the distribution for $\hat{\theta }$ values farther from $\theta_{0}$ . Seber and Wild (1989) demonstrated this same limitation for non-linear regression, coining the term “ill-conditioning” of the FI to describe the reduction in estimation precision (and associated parameter correlations) that result from non-local curvature in the likelihood surface. The full KL divergence surface leveraged in Equation (7) can accurately approximate this non-linear distribution. As a result, the KL sampling distribution allows researchers to quickly understand interactions between model parameters, how parameter estimates are affected by experimental design, and how sampling distributions might change across the parameter space, which is computationally infeasible using simulation methods for models with many parameters or with likelihoods that are numerically difficult to maximize.

## 4 Generalizable estimation and inference in the presence of parameter dependencies

**Figure 3:**
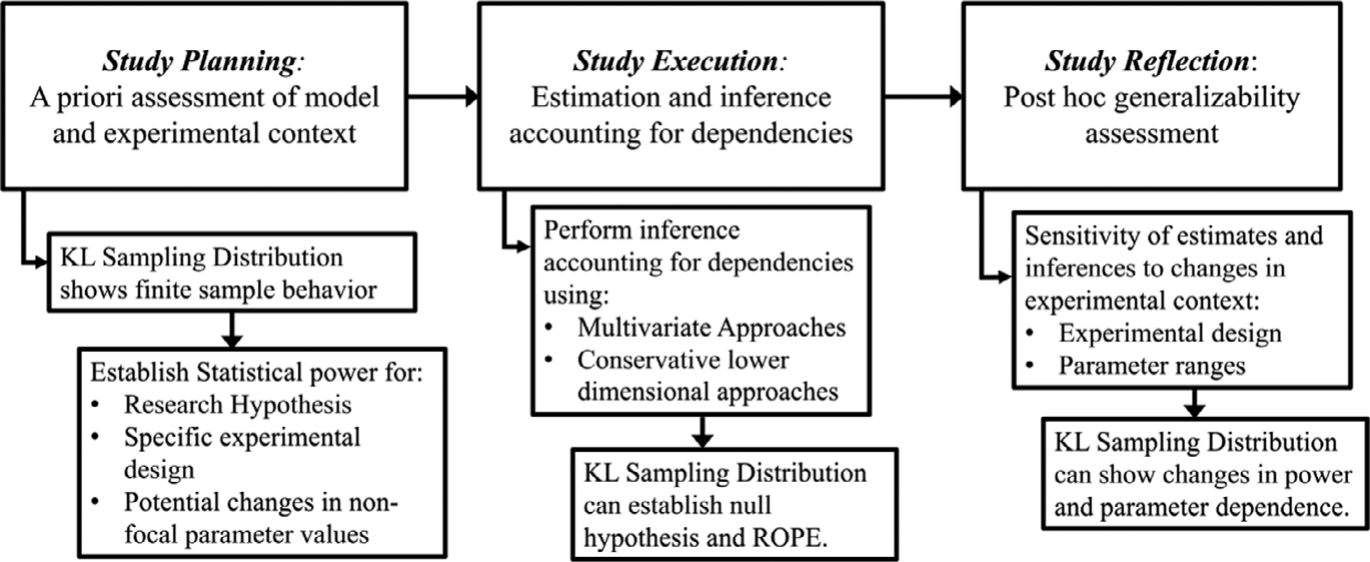
Guidance for generalizable modeling with non-linear behavioral modeling. Figure 3 long description.

For experimental research, the statistical hypothesis that a researcher plans to test determines their analysis approach and the experimental stimuli needed to collect the data to perform that analysis. We propose to look at changes in experimental context in the same way, to establish whether hypotheses based on prior work can be expected to carry over to a new set of stimuli. To help improve the generalizability of results, Figure 3 presents a schematic diagram of analyses before, during, and after data collection.

Before data collection, researchers should assess and document dependencies among model parameters to understand how these relationships influence statistical power for their research hypothesis. Power analysis should consider potential variation in statistically dependent parameters unrelated to the research hypothesis (i.e., non-focal parameters), as this will undermine generalizability. During analysis, inference methods should appropriately account for parameter dependencies. After data collection, simulation-based assessments can test the sensitivity of results to changes in experimental context, helping researchers delineate the conditions under which their findings generalize and compare outcomes across studies. Below, we discuss multiple analyses a researcher might wish to perform that leverage our novel KL sampling distribution.

### 4.1 Identifying, measuring, and documenting parameter dependencies

Stimulus- and model-driven parameter dependencies can be evaluated through visual inspection of the multivariate KL sampling distribution and summary statistics. For visual inspection, researchers can use surface plots of the marginal bivariate distributions between all pairwise combinations of the model parameters. Formally, a model is identifiable if, for all distinct parameter values, the model yields distinct probability distributions. In practice, this property is reflected in the geometry of the sampling distribution: well-identified parameters produce roughly elliptical contours with a single peak, whereas non-identifiable parameters yield maxima in at least two places or as a flat ridge across the parameter space. When the primary axes of an ellipse are tilted relative to the axes that define the parameter values (or the shape of the distribution is otherwise bent or curved), this is a visual sign of parameter association and interaction. Parameter dependencies can exist even in the absence of this visible signature of association, as even an un-tilted bivariate distribution can contain dependencies. When data are scarce and noisy, correlations can generate ridges that, while technically having a single maximum, are flat enough to affect the parameter estimates obtained via approximate optimization methods.

We use copulas to measure statistical associations and dependencies in multivariate distributions with non-parametric summary statistics (Nelsen, 2006). Copulas allow for non-parametric measures of association and dependence that can be applied to any distributional shape (see the Supplementary Material for details). For simplicity, we will use the non-parametric Spearman’s rho to measure association in our demonstrations below.

As displayed in Figure 1, the statistical dependence between estimators needs to be evaluated across different regions of the parameter space and for different types of experiments. The KL sampling distribution is computationally inexpensive and facilitates computing, storing, and visualizing summary measures for the entire parameter space and design space of a modeling study, which would be prohibitive using simulation-based sampling distributions. When fitting a behavioral model to data, we propose that researchers provide a summary of how changes to parameters or stimuli might change their results. Additionally, researchers seeking to build on past results can effectively evaluate whether the estimation properties reported in previous work would apply to their current research context (see Section 5.1 for demonstrations).

To evaluate generalizability, researchers must have some understanding of the range of parameter values that are of theoretical interest. Many models have natural bounds on parameters based on mathematically feasible values, in which case all parameter values within these bounds can be evaluated for their statistical estimation properties. Unbounded parameters can be restricted to a range that researchers expect is reasonable to detect. Alternatively, the standard approach to hypothesis testing requires specification of a null hypothesis (e.g., a parameter value that corresponds to the absence of a psychological construct), and a minimal effect size of interest that defines an alternative hypothesis to use for power calculation. As described in the next subsection, our approach follows the exact same logic, with the use of the KL sampling distribution instead of an asymptotic sampling distribution.

### 4.2 Statistical inference with parameter dependency

Null hypothesis significance testing (NHST) is widely used in the social sciences to test whether an estimate generated by a specific estimator is statistically significant. NHST methods compute the likelihood of parameter estimates as extreme as or more extreme than the current experimental estimates under a pre-specified null hypothesis. We briefly describe how KL sampling distributions can be used to test null hypotheses with statistically dependent parameter estimators.

For a model with *k* = 1 to *K* parameters, we specify a joint KL sampling distribution corresponding to the null hypothesis $\theta_{0}=\theta_{01}$ , …, $\theta_{0k}$ , …, $\theta_{0K}]$ . We test whether our parameter estimates are likely under the null hypothesis by computing their *p*-value under the joint *K*-dimensional sampling distribution for $\theta_{0}$ (the “null sampling distribution”). The *p*-value is computed by finding the likelihood of the observed data under the null sampling distribution, and then summing together the likelihood of all remaining parameter values with equal or lesser likelihood.^1^

A researcher may wish to test a subset of parameters against a null hypothesis, such as subset $\theta_{A}=\theta_{1}$ , …, $\theta_{k}]$ for *k* < *K* while accounting for their dependencies with the remaining parameters $\theta_{B}=\theta_{k+1}$ , …, $\theta_{K}]$ . In this case, we would compute our *p*-value with respect to the joint null sampling distribution for $\theta_{0}$ marginalizing across the parameters in $\theta_{B}$ . We adapt the hypothesis testing strategy outlined in Huang et al. (2024)) that finds the most conservative null hypothesis to perform an NHST in the presence of many parameters. This strategy involves identifying the most likely set of parameter values $\theta_{B}^{*}$ (with the parameters in $\theta_{A}$ constrained to their respective null values) to have generated the experimentally observed parameter estimates. This results in conservative values for the null hypothesis $\theta_{A0}=\theta_{01}$ , …, $\theta_{0k},\theta_{k+1}^{*}$ , …, $\theta_{K}^{*}]$ for testing $\theta_{A}$ because they are the closest to the observed data with respect to likelihood. Any subset of parameters can be tested using the marginal joint null distribution for the constrained parameters.

This test requires a computationally expensive search of the likelihood space, and is infeasible to perform without computationally efficient KL sampling distributions. This test is conservative, so in practice, the probability of rejecting the null when it is true is less than the theoretical type 1 error rate used to define statistical significance. Viewing NHST through signal detection theory, reduced type 1 error rates will also generate reduced statistical power. This is the cost of controlling type 1 error in the presence of parameter dependency. See Section 5.2 and Supplementary Material for the detailed computation of this hypothesis test for CPT.

### 4.3 Resampling intervals to understand variability of estimates

We can generate confidence intervals from a KL sampling distribution, which represents a resampling interval. A 95% resampling interval is the central 95% of the KL sampling distribution of the estimated parameters $\hat{\theta }$ , and represents the parameter estimates that are likely to be observed from the same experiment, assuming the estimated parameters are the true data-generating parameters. The same interval can be simulated by using a bootstrap approach, which we use in the Supplementary Material to validate the accuracy of our numerical approach.

### 4.4 Statistical power and experimental design

Each of the inferential strategies described above defines the region of the parameter space where estimated parameter values will lead the null hypothesis to be rejected (the critical region). Similar to power calculations for parametric statistical analysis, researchers can also compute the sampling distribution for any set of parameters that represent an alternative hypothesis. We compute statistical power as the probability, under the alternative hypothesis, of obtaining a parameter estimate in the critical region. Using this approach, experimenters can evaluate any given experiment (including past experiments) for statistical power and evaluate the usefulness of the experiment for their current needs. We believe this information is essential to understanding the bounds of generalizability, as this will show if estimator bias or parameter dependencies will change the interpretation of modeling results in a new experimental context and effectively reduce statistical power. As a result, researchers can evaluate any experimental design for the degree to which results from other experiments will generalize prior to collecting data.

Our methods can also be applied to experimental design more generally (Myung & Pitt, 2009). Here, the researcher starts with the cognitive model *f*(***y***; ***x***, ***θ***), and seeks out stimuli ***x*** that can produce precise parameter estimates and strong inferences. Assessing which stimuli ***x*** are the most informative in this sense requires knowledge of the sampling distributions.

### 4.5 Applicability to Bayesian inference

Our approach can also be applied to Bayesian inference. As mentioned above, posterior distributions of parameters are the result of the model, experiment, prior, and participant responses. While Bayesian inference effectively conflates these sources of dependency, sampling distributions reveal the dependencies uniquely attributable to model–stimulus interactions. Similar to the results shown in Figure 1, understanding how parameter estimation properties change across contexts is useful for evaluating the effect of priors on parameter estimation and will facilitate the interpretation of posterior distributions. Our sampling distribution can also be used to reveal designs that are more powerful at reducing uncertainty regarding parameter values. Finally, our proposed approach for summarizing the sampling distribution can be equally useful for summarizing posterior distributions obtained from an experiment or for additionally isolating the contribution of participant responses from the influence of the prior (i.e., as a form of a prior sensitivity analysis).

## 5 Reanalysis of binomial behavioral models

We perform the first comprehensive analysis of modeling dependencies for the GCM (Nosofsky, 1986) and CPT (Tversky & Kahneman, 1992) using KL sampling distributions. Each of these models is a composition of non-linear functions that induce parameter interactions and discontinuities that have reduced the generalizability of previous results (Krefeld-Schwalb et al., 2022). These reanalyses provide novel methodological insights. For example, we find that the estimation behavior of the GCM and CPT strongly depends on the experimental stimuli and requires careful study planning and study reflection to establish the generalizability of results. In the Supplementary Material, we provide additional computational details and validate the key KL sampling distributions for each model with empirically simulated sampling distributions.

*Transparency and openness*: All analyses were performed on publicly available data. All code and data used to perform these analyses are freely accessible on the open science framework (https://osf.io/9tqus/?view_only=41923e5bc75f4afca17f1046f73367ee). Analysis was performed using MATLAB version R2024a.

### 5.1 Study planning for modeling category learning behavior

The GCM (Nosofsky, 1986) predicts how people learn categorizations of stimuli based on feedback. The model parameters correspond to two psychological constructs: *w* encodes participants’ *selective attention* to various stimulus attributes used to learn the categories, and *c* encodes participants’ general *sensitivity* to attribute differences. Bartlema et al. (2014) used this model and a dataset collected by Kruschke (1993) to demonstrate how Bayesian hierarchical modeling can reveal individual differences in response strategy. Specifically, Bartlema et al. (2014) identified three different participant types, characterized by the values of *w* and *c*. However, the Kruschke (1993) study was not designed for this purpose, and it is unclear how this dataset may have affected the posterior distribution of individual differences estimated by Bartlema et al. (2014).

While this study was already run, and analyses already performed, we can evaluate how well this dataset worked for the authors’ intended purpose by taking a “study planning” perspective, as outlined in Figure 3. Our analysis reveals that the GCM induces model-driven dependencies between *w* and *c*, and that some study designs, but not others, can support the types of inferences made by Bartlema et al. (2014).

#### 5.1.1 Model and stimuli

**Figure 4:**
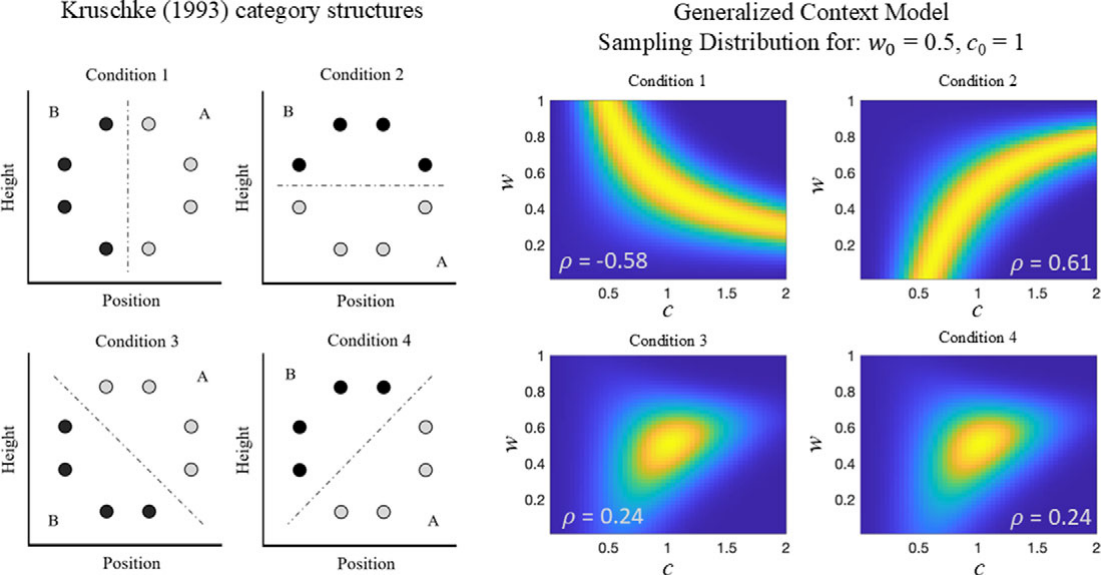
Analysis of the generalized context model. (Left) Category structure for the stimuli reproduced from Bartlema et al. (2014). (Right) KL sampling distributions for the generalized context model for each category structure in Kruschke (1993). The parameter values $w_{0}$ = 0.5 and $c_{0}$ = 1 used to make these distributions were estimated by Bartlema et al. (2014) for the full dataset. Lighter colors indicate larger probability values. Figure 4 long description.

In the Kruschke (1993) dataset, each stimulus $x_{i}$ is defined by two attributes displayed in the left panel of Figure 4: position ( $a_{i1})$ and height ( $a_{i2})$ . Participants are assigned to one of four conditions depicted in Figure 4 that differ in how these attributes relate to categories A and B.

The GCM predicts category learning using the difference between stimulus $x_{i}$ and all other stimuli $x_{j}$ , computed on each attribute separately for *m* = {1, 2} using $d_{ij}^{m}=|a_{im}-a_{jm}|$ . The model has two parameters: *w* determines the relative weight of each attribute in the category judgment; *c* is a sensitivity parameter. The GCM converts distances into a similarity measure between the stimuli given by

(8) $$\begin{array}{c} s_{ij}=e^{-c\left(wd_{ij}^{1}+\left(1-w\right)d_{ij}^{2}\right)}. \end{array}$$

The assignment of stimulus $x_{i}$ to category *A* or *B* is based on how similar the target stimulus is to the stimuli in each category. The probability that $x_{i}$ is assigned to category A is given by

(9) $$\begin{array}{c} p\left(x_{i}\;assigned\backslash kern0.17emto\backslash kern0.17emcategory\backslash ;A|w,c\right)=\frac{\sum {\backslash nolimits}_{j\in A}s_{ij}}{\sum {\backslash nolimits}_{j\in A}s_{ij}+\sum {\backslash nolimits}_{j\in B}s_{ij}}. \end{array}$$

Bartlema et al. (2014) use a Bayesian hierarchical mixture approach to identify three types of responders in the dataset based on estimates of the attention weighting and sensitivity parameters: those that attend more to position (type 1: high ***c*** and ***w***), those that attend more to height (type 2: high ***c*** and low ***w***), and random responders (type 3: low ***c***). Their analysis used uniform distributions (ranging from 0 to 1 for *w* and ranging from 0 to 5 for *c*) as uninformative prior distributions for the parameter values. In analyzing the data from condition 4 (see Figure 4), they found evidence for all three types of responders. They also analyzed the remaining conditions and concluded that condition 1 had one group of participants that attended to position, while for condition 2, they concluded that the model was not appropriate for the data based on a posterior predictive check.

#### 5.1.2 A study planning analysis

The right panel of Figure 4 shows the KL sampling distribution and predicted parameter associations for the GCM across four conditions, with $w_{0}$ and $c_{0}$ centered in the parameter space. Consistent with Nosofsky (1989) and Krefeld-Schwalb et al. (2022), GCM parameters exhibit strong associations, resulting in weak constraints on the estimated values. These associations vary dramatically with the stimuli across conditions. The KL sampling distributions reveal that the data from the four conditions are not comparable in their relationship to the model: conditions 1 and 2 induce large parameter associations of opposite sign, a problem not identified by prior work using the hierarchical Bayesian approach. When these likelihoods update a prior uniformly covering the parameter space, the resulting posteriors will differ across conditions, regardless of participants’ true parameter values. This arises because the stimuli only loosely constrain certain parameter combinations but strongly constrain others, allowing parameters near the center of the space in conditions 1 and 2 to mimic a subset of extreme values.

**Figure 5:**
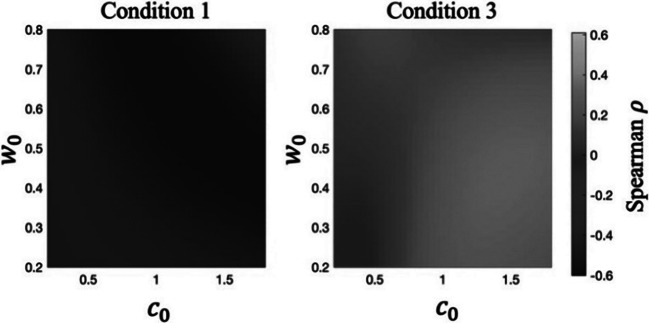
Sensitivity analysis for Spearman \unicodex3c1 between the estimators of the parameters *w* and *c* for true parameter values that span the parameter space of theoretical interest. Figure 5 long description.

Because the true participant parameters are unknown, we examined how results vary across the parameter space. We evaluated the $w_{0}$ parameter, which must be between 0 and 1, using the range 0.2–0.8 to avoid boundary effects. We evaluated the $c_{0}$ parameter, which can take any positive value, using the range 0.2–2. Figure 5 shows the estimated parameter associations (Spearman’s *ρ*) for conditions 1 and 2 across all pairwise combinations within these ranges. The two experimental conditions produce opposite parameter associations throughout the space, indicating that parameter estimates from one condition are unlikely to generalize to the other, regardless of participants’ true parameter values.

We can calculate the statistical power to distinguish between participant types for each experimental condition. Table 1 presents results using parameter values we selected to represent the participant types proposed by Bartlema et al. (2014). As indicated by the bold values in Table 1, parameter dependencies substantially reduce power to differentiate type 2 from type 3 in condition 1 and type 1 from type 3 in condition 2, whereas conditions 3 and 4 are similarly able to distinguish all three theorized participant types. Therefore, results from conditions 1 and 2 regarding estimates reflecting these types cannot be assumed to generalize to conditions 3 and 4.

**Table 1:** Statistical power for detecting individual differences based on three types of responders for each experimental condition of Kruschke (1993) Table 1 long description.

	Individual difference comparison		
	Type 1	Type 1	Type 2
	$w_{0}$ = 0.75; $c_{0}$ = 1.5	$w_{0}$ = 0.75; $c_{0}$ = 1.5	$w_{0}$ = .25; $c_{0}$ = 1.5
	Type 2	Type 3	Type 3
	$w_{0}$ = 0.25; $c_{0}$ = 1.5	$w_{0}$ = 0.5; $c_{0}$ = 0.5	$w_{0}$ = 0.5; $c_{0}$ = 0.5
Condition		Statistical power	
1	1.00	1.00	**0.09**
2	0.98	**0.24**	1.00
3	0.98	0.82	0.99
4	0.98	0.83	0.99

*Note:* Statistical power is asymmetric depending on which hypothesis is treated as the null, the results were similar with either type presented as the null, so this table presents the average.

#### 5.1.3 GCM conclusions

Overall, the parameters for the GCM are statistically dependent and highly correlated. Individual differences cannot be equally learned from the different groups of participants in this experiment because of differences in parameter dependency induced by the different stimuli shown to each group. Our analysis shows that parameter interpretation is highly contingent on stimulus design, with certain designs having very limited statistical power, a critical property of the modeling context revealed by KL sampling distributions that was not revealed by the Bartlema et al. (2014) tutorial on hierarchical Bayesian modeling.

### 5.2 Study execution and reflection for modeling risky choice behavior

CPT (Tversky & Kahneman, 1992) is a widely applied model in psychology and economics that uses non-linear and discontinuous functions to model choices that deviate from rational utility models. These deviations stem from multiple, conceptually distinct theoretical constructs, each of which is represented by a free parameter in the model. Attempting to discover which of these constructs is responsible for irrational choices has motivated many estimates of CPT parameters from behavioral data (Broomell et al., 2011; Cavagnaro et al., 2013; Donkers et al., 2001; Glöckner & Pachur, 2012; Harrison et al., 2010; Holt & Laury, 2002; Pachur et al., 2010; Rieskamp, 2008; Stott, 2006; Wu & Markle, 2008). However, CPT’s free parameters are difficult to simultaneously estimate (Nilsson et al., 2011) and appear to be highly correlated (Krefeld-Schwalb et al., 2022) due to structural dependencies between the parameters. As a result, CPT parameters can be only imprecisely measured and interpreted (Broomell & Bhatia, 2014), undermining prior attempts to explain irrational choices using CPT parameters (Krefeld-Schwalb et al., 2022; Stewart et al., 2020; Vincent & Stewart, 2020). Following the generalizable “study execution” in Figure 3, we provide the first multivariate null hypothesis significance test using KL sampling distributions to directly test CPT parameters for deviations from expected utility theory (EUT; von Neumann & Morgenstern, 1944) in the presence of parameter dependencies. Additionally, we demonstrate “study reflection” from Figure 3 by performing a post hoc power analysis to estimate the strength of the experimental design for inference. This analysis demonstrates the fragility of this experiment’s power with subtle changes in stimuli.

#### 5.2.1 Model and stimuli

The experimental stimuli are of the same type of lotteries as described in Section 2.3.3, and CPT predicts choices between two different lotteries. We adopt the CPT parameterization used in Broomell and Bhatia (2014) and Glöckner and Pachur (2012) that minimizes parameter identifiability problems. The subjective value of each lottery is determined by

(10) $$\begin{array}{c} V\left(x_{i}|\alpha ,\lambda ,\gamma \right)=v\left(x_{i1}|\alpha ,\lambda \right)*w\left(p_{i1}|\gamma \right)+v\left(x_{i2}|\alpha ,\lambda \right)*w\left(1-p_{i1}|\gamma \right). \end{array}$$

The subjective value of the outcomes, *v*(.), is given by

(11) vxij|α,λ=\lbrace_^xija\kern1.92emif\;_xij≥0−λ(−xija)\kern0.36emif\;_xij<0.

The probability of each outcome is ordered by outcome magnitude such that $x_{ij}$ < $x_{i\left(j+1\right)}$ to compute the decision weights, *w*(.), given by

(12) $$\begin{array}{c} w\left(p_{ij}|\gamma \right)=W\left(p_{ij}\cup p_{i\left(j+1\right)}\right)-W\left(p_{i\left(j+1\right)}\right)\;where\backslash ;W\left(p_{ij}\right)=e^{-{\left(-\ln\left(p_{ij}\right)\right)}^{\gamma }}. \end{array}$$

The choice probability for the gambles is given by

(13) $$\begin{array}{c} p\left(x_{1}\;prefered\backslash kern0.17emto\;x_{2}|\alpha ,\gamma ,\lambda ,\epsilon \right)=\frac{e^{\epsilon V\left(x_{1}|\alpha ,\gamma ,\lambda \right)}}{e^{\epsilon V\left(x_{1}|\alpha ,\gamma ,\lambda \right)}+e^{\epsilon V\left(x_{2}|\alpha ,\gamma ,\lambda \right)}}. \end{array}$$

This model has four parameters: α determines risk sensitivity to outcomes; γ determines probability weighting; λ determines loss aversion; and ϵ determines the sensitivity of the choice function. If we constrain γ = λ = 1, CPT reduces to a basic expected utility model.

Glöckner and Pachur (2012) collected 138 choices from 66 research participants and estimated the parameters for CPT at the individual level. They found that the out-of-sample prediction accuracy of their estimated CPT model outperformed EUT, concluding that CPT’s loss aversion and probability weighting parameters meaningfully capture the data.

#### 5.2.2 Study execution analysis

We perform a direct test of whether the estimated values [ $\hat{\alpha }$ , $\hat{\gamma }$ , $\hat{\lambda }$ , $\hat{\epsilon }]$ significantly differ from parameters that constrain CPT to behave like EUT. The CPT model has many parameter dependencies. To account for parameter dependency, we applied our conservative hypothesis testing framework introduced in Section 4.2. We computed sampling distributions for many values of $\alpha_{0}$ and $\epsilon_{0}$ under the constraint $\gamma_{0}$ = $\lambda_{0}$ = 1 and identified the distribution most likely to generate the observed estimates (see the Supplementary Material for computational details). We then tested the null hypothesis that γ≥1\;andλ≤1 against the alternative hypothesis that γ<1\;andλ>1.

**Figure 6:**
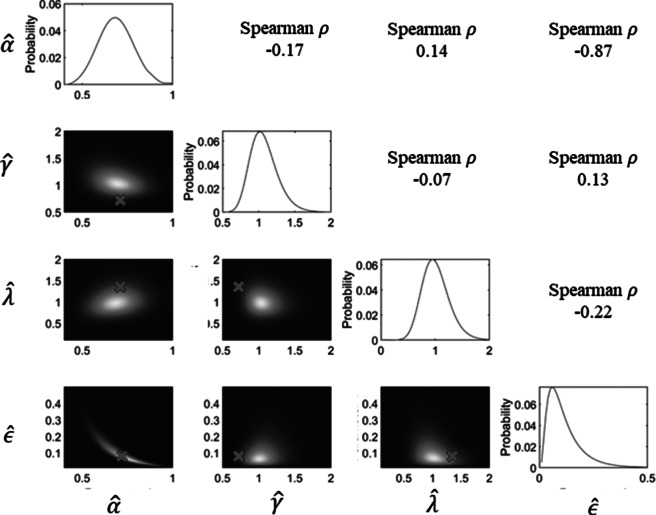
Approximate KL sampling distribution for the null hypothesis that $\alpha_{0}^{*}$ = 0.75, $\gamma_{0}$ = 1.00, ${\backslash unicodex3bb}_{0}$ = 1.00, and ${\backslash unicodex3b5}_{0}^{*}$ = 0.06 for cumulative prospect theory based on the data collected by Glöckner and Pachur (2012). Figure 6 long description.

Figure 6 displays the sampling distribution for the null hypothesis that loss aversion and probability weighting do not meaningfully capture observed choices. In each marginal bivariate plot, the estimated parameter value $\hat{\alpha }$ = 0.71, $\hat{\gamma }$ = 0.72, $\hat{\lambda }$ = 1.35, and $\hat{\epsilon }$ = 0.08 is indicated with a red “*X*.” Bivariate associations between each pair of parameters are presented above the diagonal. Lighter colors indicate larger probability values. We find that ***all*** the parameter estimates have statistical associations, reinforcing that they cannot be interpreted independently.

We tested whether the median estimate (marked with a red X) significantly differs from EUT. In computing the area under the null distribution more extreme than this estimate, we calculated a joint *p*-value for $\hat{\gamma }$ = 0.72 and $\hat{\lambda }$ = 1.35 as *p* = 0.0129, rejecting the null hypothesis that γ≥1\;andλ≤1 and that only the EUT parameters are required to model the observed choices. A post hoc power analysis using the KL sampling distribution for the estimated parameters suggests that this experiment may need more power if this were to be applied to a single decision-maker, with power = 0.55, at a 5% type 1 error rate.

This approach can also be implemented using the Bayesian Region Of Practical Equivalence (ROPE) analysis (Kruschke, 2018). For the ROPE analysis, the KL sampling distribution can define a region of equivalence around the null hypothesis that accurately accounts for structural parameter dependencies.

#### 5.2.3 Study reflection analysis

**Figure 7:**
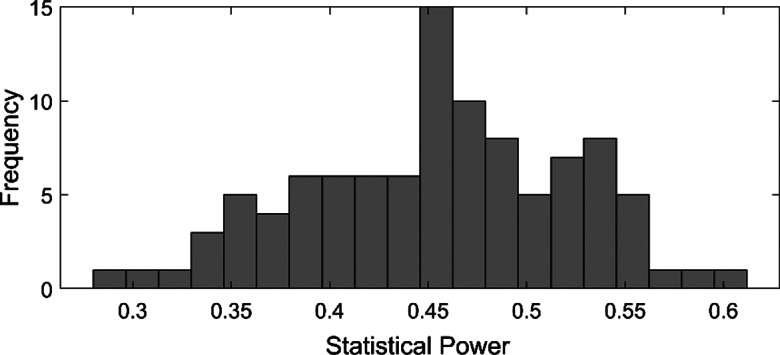
Plot of statistical power (*x*-axis) and frequency (*y*-axis) across bootstrapped CPT experiments with the same number of observed choices. Min power = 0.29, max power = 0.61. mean(power) = median(power) = 0.46.

Was this study design uniquely informative for answering their research questions? If we consider using this experiment to test the null hypothesis of H0 = [.75, 1, 1, .06] against Ha = [.71, .72, 1.35, 0.08], we get a statistical power of 0.46 at a 5% type 1 error rate. The statistical power between the exact same H0 and Ha across studies where the stimuli are randomly sampled with replacement from the original study (i.e., bootstrapped studies) is displayed in Figure 7. We observe large variability in statistical power for studies with the same number of stimuli that are randomly drawn from the same universe of potential stimuli.

#### 5.2.4 CPT conclusions

Overall, the parameters for CPT are statistically dependent and highly correlated. The study execution had limited power to detect differences between CPT and EUT. The study reflection shows that parameter interpretation and statistical power are highly contingent on task structure. In this case, inferences from one study can be difficult to replicate, even when evaluating the exact same participants with a random sample of the same stimuli.

## 6 Discussion

The study of social and cognitive science can be greatly advanced by using behavioral models to precisely specify theories and summarize datasets. As models grow increasingly complex, the relationship between model parameters and experimental data becomes more difficult to understand. In contexts where this relationship is impossible to understand, both Bayesian and frequentist modeling results can be inadvertently driven by aspects of the experimental stimuli, and the results of statistical analyses can fail to replicate in experiments with slightly different stimuli (e.g., see the analysis of the Description–Experience Gap presented in Broomell and Bhatia, 2014). More critically, such non-generalizable inferences can lead to inaccurate conclusions and predictions about behavior in real-world contexts with only minor differences in the experimental stimuli. This issue is not limited to psychological research and affects many areas of scientific study. When seemingly unimportant changes to the models or to the stimuli used to generate responses result in failures to replicate or generalize, it can be difficult, if not impossible, to diagnose the source of the problem. Our proposed framework provides researchers with a systematic approach to diagnose these failures in past studies and prevent them from occurring in future studies by following the recommendations in Figure 3.

We introduce a computationally efficient mathematical tool to numerically approximate sampling distributions for binomial non-linear and discontinuous behavioral models that accurately capture finite sample behavior. Using contemporary behavioral models, we demonstrate the application of our approach and test its validity while also demonstrating how to evaluate estimates from different datasets, understand dynamically changing estimation bias, variance, and dependence for parameters, and perform hypothesis tests and power analyses for complex non-linear behavioral models. With our proposed approach, researchers can understand how estimation properties change as a function of experimental design and evaluate the strength of experimental stimuli for model estimation and testing prior to data collection, all of which are required to understand the generalizability of modeling results in the presence of parameter dependencies.

With current analysis and reporting practices, researchers, reviewers, and editors are blind to the interplay between complex models and the data. As a result, they are unable to evaluate the accuracy and generalizability of scientific inferences regarding model parameters (Broomell et al., 2019). Our approach both reveals the statistical dependencies of parameter estimates and facilitates accurate inferences in the presence of these dependencies. As demonstrated by the behavioral models analyzed in this article, there are many ways in which the generalizability of results from non-linear models can be limited by seemingly subtle changes to the stimuli. This framework is the first attempt to clarify the limits of generalizability in this space.

In the development and application of our approach, we have also contributed to theory in two areas of behavioral modeling that have been clouded by statistical dependence (Krefeld-Schwalb et al., 2022). First, we analyze a foundational model of category learning, the GCM (Nosofsky, 1986). We find that the statistical properties of the parameter estimators for the GCM can change wildly depending on the experimental stimuli used to elicit responses from participants. The effects of experimental stimuli on modeling results are large enough that even uninformative prior beliefs that the parameters are uniformly distributed across the parameter space can result in vastly different posterior distributions for otherwise identical experimental participants.

Second, we analyzed CPT (Tversky & Kahneman, 1992), one of the most widely applied models for predicting and explaining risky choice behavior in economics and psychology. We establish moderate statistical power for a current study (Glöckner & Pachur, 2012), and find it is unlikely that other similar experiments can test EUT with the same degree of confidence as the experiment conducted by Glöckner and Pachur (2012). Future tests of this result should establish whether a particular set of choices has the potential to lead to a significant result or not, and clearly quantify the statistical power.

Finally, our analysis also demonstrates an important limitation to Bayesian analysis, which has increasingly enabled researchers to fit more complex behavioral models. If Bayesian analysis is applied to models with unknown statistical properties and dependencies between parameters, these unknown properties can interact with prior distributions to alter the posterior distribution of parameter values in ways that were not intended by the researcher. Additionally, posterior distributions do not incorporate uncertainty about how the results might change if the experimental design changed. Therefore, our framework is also beneficial for Bayesian modeling approaches by helping researchers understand how the experimental design can affect the separate identifiability of parameters given scarce and noisy data.

In sum, we developed and validated our approach for two types of models: those with Gaussian and Binomial likelihoods. Our approach has facilitated completely novel analyses of binomial models by introducing numerically simple computations of their sampling distributions that were inaccessible to prior work. Our approach has great promise to be extended to other types of likelihoods, for example, to models with multinomial distributions, beta distributions, curved exponential families, and even models with predictive distributions that can only be simulated. This novel approach can potentially be applied to other areas of statistical inference, for example, to help researchers clarify the effects of violations of independence for ANOVAs applied to unequal sample sizes and regression results with correlated predictors. With future research, we hope that a broader application of this approach can further facilitate generalizable and interpretable results from behavioral modeling, underpinning stronger and more robust psychological theories and predictions.

## Supporting information

10.1017/psy.2026.10089.sm001 Broomell et al. supplementary material Broomell et al. supplementary material

## Footnotes

1
Similar to the omnibus test of an ANOVA, rejecting H0 can only be interpreted as having found that at least one parameter that is unlikely given the null hypothesis.

## Supplementary material

To view supplementary material for this article, please visit http://doi.org/10.1017/psy.2026.10089.

## Figure 1 Long description

The grid contains six scatterplots arranged in two rows and three columns. The top row shows baseline simulations, the bottom row shows increased variance conditions. The left two columns plot b hat sub 2 versus b hat sub 1 for linear regression models: the first column is labeled Linear Regression with Stimulus Dependency, the second column is labeled Linear Regression with Structural Dependency. The rightmost column plots epsilon hat versus alpha hat for a Non-linear Model with Structural Dependency. In each panel, four distinct clusters of points are color-coded: black, red, green, and blue. In the top row, increased variance in x sub 1 (left and middle columns) or payoff values (right column) leads to greater spread and overlap among clusters, especially for red and green. In the bottom row, clusters are more compact and separated. Axis ranges are b hat sub 1 and b hat sub 2 from 0 to 4, alpha hat from 0 to 2.5, epsilon hat from 0 to 2.5. Color coding corresponds to parameter values: black is 1, 1 or 0.3, 0.2; red is 1, 3 or 0.3, 1; green is 3, 1 or 1, 0.2; blue is 3, 3 or 1, 1.

Navigate back to Figure 1.

## Figure 2 Long description

On the left, the Gaussian model panel shows a 3D surface plot of KL divergence with axes labeled Mean and Variance, and a marked curvature at theta sub 0. An arrow labeled ‘Convert surface to probability Eq. 7’ points downward to three plots: the leftmost is a heatmap labeled Asymptotic sampling distribution with axes Mean and Variance, showing a symmetric central peak; the center is a scatter plot labeled Simulated sampling distribution with axes Est. Mean and Est. Variance, showing a dense central cluster; the rightmost is a heatmap labeled KL sampling distribution, visually matching the asymptotic distribution. On the right, the nonlinear behavioral model panel shows a 3D KL divergence surface with axes Parameter 1 and Parameter 2, and a marked curvature at theta sub 0. An arrow labeled ‘Convert surface to probability Eq. 7’ points downward to three plots: the leftmost is a heatmap labeled Asymptotic sampling distribution with axes Parameter 1 and Parameter 2, showing an elliptical peak; the center is a scatter plot labeled Simulated sampling distribution with axes Est. Param. 1 and Est. Param. 2, showing a curved cluster; the rightmost is a heatmap labeled KL sampling distribution, closely matching the simulated distribution. Lighter colors indicate higher values in all heatmaps.

Navigate back to Figure 2.

## Figure 3 Long description

From left to right, the flowchart has three main columns. The first column is Study Planning, labeled as a priori assessment of model and experimental context. Below, an arrow points to KL Sampling Distribution shows finite sample behavior, which leads to Establish Statistical power for: Research Hypothesis, Specific experimental design, Potential changes in non-focal parameter values. The second column is Study Execution, labeled as estimation and inference accounting for dependencies. Below, an arrow points to Perform inference accounting for dependencies using: Multivariate Approaches, Conservative lower dimensional approaches, which leads to KL Sampling Distribution can establish null hypothesis and R O P E. The third column is Study Reflection, labeled as post hoc generalizability assessment. Below, an arrow points to Sensitivity of estimates and inferences to changes in experimental context: Experimental design, Parameter ranges, which leads to KL Sampling Distribution can show changes in power and parameter dependence. All columns are connected sequentially by rightward arrows, forming a continuous process.

Navigate back to Figure 3.

## Figure 4 Long description

There are four panels in two columns. The left column shows scatter plots labeled Condition 1 to Condition 4, each with axes Height and Position. Black circles (category B) and white circles (category A) are separated by dashed lines, which are vertical in Condition 1, horizontal in Condition 2, diagonal from top-left to bottom-right in Condition 3, and diagonal from bottom-left to top-right in Condition 4. The right column shows heatmaps for the generalized context model, with axes w (vertical, 0 to 1) and c (horizontal, 0.5 to 2). Each heatmap corresponds to the scatter plot on its left. Lighter colors indicate higher probability. Condition 1 has a curved band with negative correlation (rho equals minus 0.58), Condition 2 has a curved band with positive correlation (rho equals 0.61), Conditions 3 and 4 have central peaks with low correlation (rho equals 0.24). Parameter values w sub 0 equals 0.5 and c sub 0 equals 1 are noted above the heatmaps.

Navigate back to Figure 4.

## Figure 5 Long description

The left panel labeled Condition 1 shows a heatmap with c sub 0 on the x axis from 0.2 to 1.8 and w sub 0 on the y axis from 0.2 to 0.8. The color is uniformly dark blue, indicating Spearman rho values near negative 0.6. The right panel labeled Condition 3 uses the same axes. The color transitions from blue at the lower left to green and yellow at the upper right, indicating Spearman rho values from negative 0.6 to positive 0.6. The color bar to the right of the panels is labeled Spearman rho, with a gradient from blue at negative 0.6 through green at zero to yellow at positive 0.6.

Navigate back to Figure 5.

## Table 1 Long description

The table is organized with columns for individual difference comparisons: Type 1 versus Type 1, Type 1 versus Type 2, Type 2 versus Type 3. The first parameter row lists w sub 0 equals 0.75 and c sub 0 equals 1.5 for both Type 1 versus Type 1 and Type 1 versus Type 2, and w sub 0 equals 0.25 and c sub 0 equals 1.5 for Type 2 versus Type 3. The next row lists w sub 0 equals 0.25 and c sub 0 equals 1.5 for Type 2 versus Type 1, and w sub 0 equals 0.5 and c sub 0 equals 0.5 for both Type 3 versus Type 2 and Type 3 versus Type 3. The lower section lists four experimental conditions in the leftmost column. For Condition 1, statistical power is 1.00 for Type 1 versus Type 1, 1.00 for Type 1 versus Type 2, and 0.09 for Type 2 versus Type 3. For Condition 2, power is 0.98 for Type 1 versus Type 1, 0.24 for Type 1 versus Type 2, and 1.00 for Type 2 versus Type 3. For Condition 3, power is 0.98 for Type 1 versus Type 1, 0.82 for Type 1 versus Type 2, and 0.99 for Type 2 versus Type 3. For Condition 4, power is 0.98 for Type 1 versus Type 1, 0.83 for Type 1 versus Type 2, and 0.99 for Type 2 versus Type 3. The note below the table states that statistical power is asymmetric depending on which hypothesis is treated as the null, but the table presents the average as results were similar either way.

Navigate back to Table 1.

## Figure 6 Long description

From top left to bottom right, the matrix rows and columns are labeled alpha hat, gamma hat, lambda hat, and epsilon hat. Diagonal cells display marginal probability distributions as line graphs for each parameter. Off-diagonal cells show joint distributions as colored heatmaps, with axes corresponding to the parameter pair and a red X marking the mode. Above or to the right of each off-diagonal cell, the Spearman rho value quantifies the correlation between the parameter pair: alpha hat and gamma hat negative 0.17, alpha hat and lambda hat 0.14, alpha hat and epsilon hat negative 0.87, gamma hat and lambda hat negative 0.07, gamma hat and epsilon hat 0.13, lambda hat and epsilon hat negative 0.22. Marginal distributions are unimodal and symmetric except for epsilon hat, which is right-skewed. Joint distributions show varying degrees of elliptical spread, with the strongest negative correlation between alpha hat and epsilon hat.

Navigate back to Figure 6.
