# Supplementary material for: Toward Generalizable Estimation of Behavioral Models with Parameter Dependencies
Source: Psychometrika. 2026 Feb 6;91(2):473–93. doi: 10.1017/psy.2026.10089 (PMC13294615; doi:10.1017/psy.2026.10089)
Supplement: Broomell et al. supplementary material [file S0033312326100891sup001.docx]

Supplemental Materials

The Generalizability of Behavioral Modeling with Parameter Dependencies

1/22/2026

**Introduction to Copulas**

Given a bivariate distribution *f*(*x*,*y*), Nelson (2006) defines a copula as a function *C*(*u*,*v*) such that *C*(*u*,0) = *C*(0,*v*) = 0, *C*(*u*,1) = *u*, *C*(1,*v*) = *v*, and for every $u_{1}$, $u_{2}$, $v_{1}$, $v_{2}$ in [0,1],

$$C\left( u_{2},v_{2} \right)-C\left( u_{2},v_{1} \right)-C\left( u_{1},v_{2} \right)+C\left( u_{1},v_{1} \right)\geq0.$$

The variable *u* is equal to the marginal cumulative distribution function of *x*, and *v* is equal to the marginal cumulative distribution function of *y*. With this definition, the copula function *C*(*u*,*v*) represents the joint structure of the distributions *x* and *y* after removing the marginal distributions of *x* and *y*.

Following Nelson (2006), we can compute a measure of association directly from the copula based on Spearman’s rho,

$$\rho_{xy}^{SP}=12\iint\left[ C\left( u,v \right)-u*v \right]dudv, (4)$$

and a measure of dependence based on Schweizer and Wolf’s sigma,

$$\sigma_{xy}^{SW}=12\iint|C\left( u,v \right)-u*v|dudv. (5)$$

Spearman’s rho can range from [-1, 1] and can be interpreted in the same way as a standard correlation coefficient. Schweizer and Wolf’s sigma ranges from 0 (no dependency) to 1 (complete dependency). These measures can be computed for each bivariate marginal (or even conditional) distribution for all pairwise combinations of the model parameters.

**Analytical Derivation of the KL Sampling Distribution**

The matrix ***x*** contains *s* columns the define the stimuli across *n* experimental trials. The vector ***y*** contains *n* behavioral responses to the stimuli in ***x***. A behavioral model defines the function *f*(y; ***x***|***θ***) = ***Y*** where the vector ***θ*** contains the free model parameters that define how the stimuli ***x*** are converted into the predictive distributions ***Y =*** *p*(***y***|***x,θ***) for each of the *n* statistically independent observations. The likelihood function for the model parameters is defined by the behavioral model and the stimuli, expressed as *L*(***θ***|***x***,***y***) = *p*(***y***|***x***,***θ***).

Kullback-Leibler (KL) divergence is a measure of relative entropy from a focal probability distribution to an alternative probability distribution, which is quantified as the additional bits (or nats) of information required to describe the focal distribution when assuming an alternative distribution (Cover & Thomas, 1991; Kullback & Leibler, 1951). As such, this is a measure of the distance from a focal distribution $p_{0}$ to the alternative distribution $p_{1}$ in units of entropy, expressed as $D_{KL}(p_{0}||p_{1})$ where the double bars indicate divergence.

The KL divergence of the likelihood of the focal parameter, $\boldsymbol{\theta}_{\boldsymbol{0}}$, to the likelihood defined by an alternative parameter set, $\boldsymbol{\theta}_{\boldsymbol{1}}$, is given by,

$$D_{KL}\left( L(\boldsymbol{\theta}_{\boldsymbol{0}}|\boldsymbol{x},\boldsymbol{y})||L(\boldsymbol{\theta}_{\boldsymbol{1}}|\boldsymbol{x},\boldsymbol{y}) \right)= E_{p({\boldsymbol{y}\boldsymbol{|}\boldsymbol{\theta}}_{\boldsymbol{0}},\boldsymbol{x})}\left[ \ln\left( \frac{p\left( \boldsymbol{y} | \boldsymbol{\theta}_{\boldsymbol{0}},\boldsymbol{x} \right)}{p\left( \boldsymbol{y} | \boldsymbol{\theta}_{\boldsymbol{1}}\boldsymbol{x} \right)} \right) \right]. (S1)$$

Assuming each individual observation *y_i_* is independent, Eq S1 can be broken down into,

$$D_{KL}\left( L(\boldsymbol{\theta}_{\boldsymbol{0}}|\boldsymbol{x},\boldsymbol{y})||L(\boldsymbol{\theta}_{\boldsymbol{1}}|\boldsymbol{x},\boldsymbol{y}) \right)= \sum_{i}^{n} D_{KL}\left( L(\boldsymbol{\theta}_{\boldsymbol{0}}|\boldsymbol{x}_{i\cdot},y_{i})||L(\boldsymbol{\theta}_{\boldsymbol{1}}|\boldsymbol{x}_{i\cdot},y_{i}) \right). (S2)$$

We compute a surface where each point represents the KL divergence between $\boldsymbol{\theta}_{\boldsymbol{0}}$ (the focal parameter value) from all alternative parameters $\boldsymbol{\theta}_{\boldsymbol{1}}\in\boldsymbol{\Theta}$. The KL divergence surface will equal zero at $\boldsymbol{\theta}_{\boldsymbol{1}}$ = $\boldsymbol{\theta}_{\boldsymbol{0}}$. If there exists another alternative parameter in the set ***Θ*** (that is not equal to $\boldsymbol{\theta}_{\boldsymbol{0}}$) with a KL divergence of zero, then the model will meet the definition of being unidentifiable (Lehmann & Casella, 1998). The rate at which the KL divergence surface increases as the parameters move away from $\boldsymbol{\theta}_{\boldsymbol{0}}$ indicates the degree to which the alternative parameters generate different likelihoods. Steeper increases in the KL surface means that the estimation variance of the parameter can be lower. The surface has the same number of dimensions as the number of parameters in ***θ***, and can capture how parameters interact as well as parameter combinations that can mimic other sets of parameters.

**Transforming the KL surface into a Sampling Distribution**

To transform the KL surface into a sampling distribution, the probability of estimating each parameter value in the set Θ is inversely proportional to the KL divergence. KL divergence is on a log scale, so we start by taking the exponential of the KL divergence between the likelihoods to convert log likelihoods back to a probability scale given by,

$$p\left( \hat{\boldsymbol{\theta}}= \boldsymbol{\theta}_{1} | \boldsymbol{x} \right)\propto\exp\left[ -1*D_{KL}(L(\boldsymbol{\theta}_{0} |\boldsymbol{x,y)||}L(\boldsymbol{\theta}_{1}│\boldsymbol{x,y)}) \right]. (S3)$$

However, the measure of KL divergence is not identical to likelihood of estimation, and we will need to solve for corrections to make this distribution match the true sampling distribution more closely. We derive two such corrections: a scale correction, and a distributional correction.

***Scale Correction***. When the likelihood surface is defined by an independent location and scale parameter (such as a normal Gaussian distribution), the KL divergence surface will show a dependence between the location parameter and the scale parameter. This is because the probability distribution of the location parameter will depend on the scale parameter. For example, consider a normal distribution with location defined by the mean *μ* and scale defined by the variance $\sigma^{2}$. When variance is smaller (larger), two means will generate likelihoods that are more (less) divergent. However, this dependence is not reflected in the sampling distribution of the Gaussian mean and variance parameters (Hogg & Craig, 1965). When estimating a mean and variance from data, the chances of observing a certain mean does not change due to the estimated variance parameter. Therefore, the KL divergence surface needs to be corrected to approximate the statistical properties of the sampling distribution more closely. This correction is performed in two steps: (a) multiplying the KL divergence by the scaling parameter of the alternative parameter set $\sigma_{1}^{2}$ and then (b) dividing the KL divergence by the scaling parameter of the focal parameter set $\sigma_{0}^{2}$. This results in the probability of estimating $\boldsymbol{\theta}=[\mu, \sigma^{2}]$ as given by,

$$p\left( \hat{\boldsymbol{\theta}}= \boldsymbol{\theta}_{1}=[\mu_{1}, \sigma_{1}^{2}] | \boldsymbol{x,y} \right)\propto\exp\left[ -1*\frac{\sigma_{1}^{2}}{\sigma_{0}^{2}}*D_{KL}(L(\boldsymbol{\theta}_{0}|\boldsymbol{x,y)||}L(\boldsymbol{\theta}_{1}|\boldsymbol{x,y)}) \right]. (S4)$$

We define the scale correction to account for distributional dependence of independent scale and location parameters with $\alpha= \sigma_{1}^{2}/\sigma_{0}^{2}$.

***Distributional Correction.*** The theoretical asymptotic sampling distribution for the parameters of the Gaussian distribution are derived using Fisher Information. Fisher information can be expressed as the local curvature (second derivative) of the KL divergence of the likelihood function at $\boldsymbol{\theta}_{\boldsymbol{0}}$. For parameters $\theta_{i}$ and $\theta_{j}$ contained in $\boldsymbol{\theta}$, Fisher information is derived from the derivative $I\left( \theta_{i},\theta_{j} \right)=\frac{\partial^{2}}{\partial\theta_{i}\partial\theta_{j}}D_{KL}(p_{\theta_{0}}|\left| p_{\theta} \right)\left| { \atop\theta=\theta_{0}} \right..$ The theoretical asymptotic sampling distribution for the Gaussian mean and variance are then,

$$f\left( \hat{\mu}= \mu_{1} | \mu_{0}, \sigma_{0}^{2}/n \right)= \frac{1}{\sqrt{2\pi\sigma_{0}^{2}/n}}\exp\left( \frac{-\left( \mu_{1}- \mu_{0} \right)^{2}}{2\sigma_{0}^{2}/n} \right) (S5)$$

$$f\left( \hat{\sigma}^{2}= \sigma_{1}^{2} | \sigma_{0}^{2}, \left( {2\sigma}_{0}^{2} \right)^{2}/n \right)= \frac{1}{\sqrt{2\pi\left( 2\sigma_{0}^{2} \right)^{2}/n}}\exp\left( \frac{-\left( \sigma_{1}^{2}- \sigma_{0}^{2} \right)^{2}}{\left( 2\sigma_{0}^{2} \right)^{2}/n} \right) (S6)$$

The variance terms in these distributions are the inverse of the corresponding elements of the Fisher information matrix derived from the KL divergence. These two parameters are independent of each other, so the joint distribution of both parameters is equal to the product of Eq. S5 and S6.

We can derive the analytical solution of Eq. S4 with likelihoods based on sampling *n* samples from a normal Gaussian distribution. We can therefore solve for exactly how close our KL divergence-based sampling distribution comes to the theoretical sampling distribution by comparing the analytical solution of our KL divergence approach to the sampling distribution formed by Eq. S5 and S6. Comparing these two analytical solutions for equality will reveal any additional corrections needed to make the KL divergence surface equal to the theoretical sampling distribution for the normal Gaussian distribution.

Let $p_{0}(y) \sim N(\mu_{0},\sigma_{0}^{2})$ and $p_{1}(y) \sim N(\mu_{1},\sigma_{1}^{2})$. First, we derive the solution for the KL divergence between these two normal distributions as follows:

$$D_{KL}(p_{0}(y)||p_{1}\left( y \right))= \int p_{0}\left( y \right)\ln\left( \frac{p_{0}\left( y \right)}{p_{1}\left( y \right)} \right)dy= \int p_{0}\left( y \right) \left[ \ln\left( p_{0}\left( y \right)-\ln\left( p_{1}\left( y \right) \right) \right) \right]dy$$

$$= \int p_{0}\left( y \right)\ln\left( p_{0}\left( y \right) \right)dy- \int p_{0}\left( y \right)\ln\left( p_{1}\left( y \right) \right)dy$$

$$= \left[ -\frac{1}{2}\ln\left( 2\pi\sigma_{0}^{2} \right)-\frac{1}{2} \right]- \left[ -1\left( \frac{1}{2}\ln\left( 2\pi\sigma_{1}^{2} \right)+ \frac{\sigma_{0}^{2}+\left( \mu_{0}- \mu_{1} \right)^{2}}{2\sigma_{1}^{2}} \right) \right]$$

$$=\ln\left( \frac{\sigma_{1}}{\sigma_{0}} \right)+ \frac{\sigma_{0}^{2}+\left( \mu_{0}- \mu_{1} \right)^{2}}{2\sigma_{1}^{2}}-\frac{1}{2}. (S7)$$

Second, we derive the solution for Eq. S4 using Eq. S7. Assuming we have *n* observations, we can leverage Eq. S2 to get the KL divergence for the full sample as the sum of *n* divergences given by,

$$p\left( \hat{\boldsymbol{\theta}}= \boldsymbol{\theta}_{1}=[\mu_{1}, \sigma_{1}^{2}] | \boldsymbol{x}\boldsymbol{,}\boldsymbol{y} \right)\propto\exp\left( -n*\frac{\sigma_{1}^{2}}{\sigma_{0}^{2}}*D_{KL}\left( p_{0}(y)||p_{1}\left( y \right) \right) \right)=$$

$$= exp\left( -n*\frac{\sigma_{1}^{2}}{\sigma_{0}^{2}}*\left[ \ln\left( \frac{\sigma_{1}}{\sigma_{0}} \right)+ \frac{\sigma_{0}^{2}+\left( \mu_{0}- \mu_{1} \right)^{2}}{2\sigma_{1}^{2}}-\frac{1}{2} \right] \right)$$

$$=\exp\left( -n*\frac{\sigma_{1}^{2}}{\sigma_{0}^{2}}\ln\left( \frac{\sigma_{1}}{\sigma_{0}} \right) \right)\exp\left( \frac{n\sigma_{1}^{2}}{2\sigma_{0}^{2}}-\frac{n}{2} \right)\exp\left( \frac{-\left( \mu_{0}- \mu_{1} \right)^{2}}{2\sigma_{0}^{2}/n} \right). (S8)$$

The last exponential term in the last line of Eq. S8 is equal to $f\left( \hat{\mu}= \mu_{1} | \mu_{0}, \sigma_{0}^{2}/n \right)*\sqrt{2\pi\sigma_{0}^{2}/n}$, so we can make that substitution. The values for $\sigma_{0}^{2}$, $\mu_{0}$, and *n* are constant and do not affect the probability, so we place all constant values into *c* to shorten the equation to get,

$$p\left( \hat{\boldsymbol{\theta}}= \boldsymbol{\theta}_{1}=[\mu_{1}, \sigma_{1}^{2}] | \boldsymbol{x,y} \right)\propto\exp\left( -n*\frac{\sigma_{1}^{2}}{\sigma_{0}^{2}}\ln\left( \frac{\sigma_{1}}{\sigma_{0}} \right) \right)\exp\left( \frac{n\sigma_{1}^{2}}{2\sigma_{0}^{2}}-\frac{n}{2} \right) f\left( \hat{\mu}= \mu_{1} | \mu_{0},\sigma_{0}^{2}/n \right)*c. (S9)$$

Next, we add in the asymptotic sampling distribution of $\hat{\sigma}^{2}$ along with its inverse,

$$p\left( \hat{\boldsymbol{\theta}}= \boldsymbol{\theta}_{1}=[\mu_{1}, \sigma_{1}^{2}] | \boldsymbol{x,y} \right)\propto\exp\left( -n*\frac{\sigma_{1}^{2}}{\sigma_{0}^{2}}\ln\left( \frac{\sigma_{1}}{\sigma_{0}} \right) \right)\exp\left( \frac{n\sigma_{1}^{2}}{2\sigma_{0}^{2}}-\frac{n}{2} \right)f\left( \hat{\mu}= \mu_{1} | \mu_{0},\frac{\sigma_{0}^{2}}{n} \right)*c*\exp\left( \frac{\left( \sigma_{1}^{2}- \sigma_{0}^{2} \right)^{2}}{\frac{\left( 2\sigma_{0}^{2} \right)^{2}}{n}} \right)\exp\left( \frac{{-\left( \sigma_{1}^{2}- \sigma_{0}^{2} \right)}^{2}}{\frac{\left( 2\sigma_{0}^{2} \right)^{2}}{n}} \right). (S10)$$

Similar to above, the last term of Eq. S10 is equal to $f\left( \hat{\sigma}^{2}= \sigma_{1}^{2} | \sigma_{0}^{2}, \left( {2\sigma}_{0}^{2} \right)^{2}/n \right)*\sqrt{2\pi\left( 2\sigma_{0}^{2} \right)^{2}/n}$, so we make another substitution for this term adding to the constant term *c* the constant parts of the substitution.

$$p\left( \hat{\boldsymbol{\theta}}= \boldsymbol{\theta}_{1}=[\mu_{1}, \sigma_{1}^{2}] | \boldsymbol{x,y} \right)\propto\exp\left( -n*\frac{\sigma_{1}^{2}}{\sigma_{0}^{2}}\ln\left( \frac{\sigma_{1}}{\sigma_{0}} \right) \right)\exp\left( \frac{n\sigma_{1}^{2}}{2\sigma_{0}^{2}}-\frac{n}{2} \right)f\left( \hat{\mu}= \mu_{1} | \mu_{0},\frac{\sigma_{0}^{2}}{n} \right)f\left( \hat{\sigma}^{2}= \sigma_{1}^{2} | \sigma_{0}^{2},\frac{\left( {2\sigma}_{0}^{2} \right)^{2}}{n} \right)*c*\exp\left( \frac{\left( \sigma_{1}^{2}- \sigma_{0}^{2} \right)^{2}}{\frac{\left( 2\sigma_{0}^{2} \right)^{2}}{n}} \right). (S11)$$

Consolidating the terms in the exponential gives us a shorter term,

$$\exp\left( -n*\frac{\sigma_{1}^{2}}{\sigma_{0}^{2}}\ln\left( \frac{\sigma_{1}}{\sigma_{0}} \right)+\frac{n\sigma_{1}^{2}}{2\sigma_{0}^{2}}-\frac{n}{2}+\frac{\left( \sigma_{1}^{2}- \sigma_{0}^{2} \right)^{2}}{\left( 2\sigma_{0}^{2} \right)^{2}/n} \right)=\exp\left( -n*\frac{\sigma_{1}^{2}}{\sigma_{0}^{2}}\ln\left( \frac{\sigma_{1}}{\sigma_{0}} \right)+ \frac{{{(\sigma}_{1}^{2})}^{2} - {(\sigma}_{0}^{2})^{2}}{\left( 2\sigma_{0}^{2} \right)^{2}/n} \right). (S12)$$

Substituting Eq. S12 into Eq. S11 gives the final analytical solution for Eq. S4 as a function of the asymptotic sampling distributions of the mean and variance of the normal Gaussian distribution,

$$p\left( \hat{\boldsymbol{\theta}}= \boldsymbol{\theta}_{1}=[\mu_{1}, \sigma_{1}^{2}] | \boldsymbol{x,y} \right)\propto\exp\left( -n*\frac{\sigma_{1}^{2}}{\sigma_{0}^{2}}\ln\left( \frac{\sigma_{1}}{\sigma_{0}} \right)+ \frac{{{(\sigma}_{1}^{2})}^{2} - {(\sigma}_{0}^{2})^{2}}{\frac{\left( 2\sigma_{0}^{2} \right)^{2}}{n}} \right)f\left( \hat{\mu}= \mu_{1} | \mu_{0},\frac{\sigma_{0}^{2}}{n} \right)*f\left( \hat{\sigma}^{2}= \sigma_{1}^{2} | \sigma_{0}^{2},\frac{\left( {2\sigma}_{0}^{2} \right)^{2}}{n} \right)*c. (S13)$$

Therefore, we have solved for the exact transformation of the KL divergence surface to generate the asymptotic sampling distribution for the normal distribution as the following,

$$p_{KL}\left( \hat{\boldsymbol{\theta}}= \boldsymbol{\theta}_{1}=[\mu_{1}, \sigma_{1}^{2}] | \boldsymbol{x}\boldsymbol{,}\boldsymbol{y} \right)=c*\exp\left[ -1*\frac{\sigma_{1}^{2}}{\sigma_{0}^{2}}*D_{KL}(L(\boldsymbol{\theta}_{0}|\boldsymbol{x}\boldsymbol{,}\boldsymbol{y}\boldsymbol{)||}L(\boldsymbol{\theta}_{1}|\boldsymbol{x}\boldsymbol{,}\boldsymbol{y}\boldsymbol{)}) \right]/\exp\left[ -n*\frac{\sigma_{1}^{2}}{\sigma_{0}^{2}}\ln\left( \frac{\sigma_{1}}{\sigma_{0}} \right)+ \frac{{{(\sigma}_{1}^{2})}^{2} - {(\sigma}_{0}^{2})^{2}}{\frac{\left( 2\sigma_{0}^{2} \right)^{2}}{n}} \right] =f\left( \hat{\mu}= \mu_{1} | \mu_{0},\frac{\sigma_{0}^{2}}{n} \right)*f\left( \hat{\sigma}^{2}= \sigma_{1}^{2} | \sigma_{0}^{2},\frac{\left( {2\sigma}_{0}^{2} \right)^{2}}{n} \right). (S14)$$

The expression in the second exponential term in Eq. S14 defines how the KL divergence surface differs from the asymptotic sampling distribution for the mean and variance of a normal Gaussian distribution. We define our second correction to adjust the KL distribution to match the sampling distribution as $\beta= -n*\frac{\sigma_{1}^{2}}{\sigma_{0}^{2}}\ln\left( \frac{\sigma_{1}}{\sigma_{0}} \right)+ \frac{{{(\sigma}_{1}^{2})}^{2} - {(\sigma}_{0}^{2})^{2}}{\left( 2\sigma_{0}^{2} \right)^{2}/n}$.

We explore further the mechanics of the correction $\beta$. The variables in the expression do not contain any of the mean terms, so the correction is accounting for differences regarding the variance terms. To get a better sense of what the $\beta$ correction does, Figure S1 plots the $\beta$ correction as a percent change from the asymptotic distribution to the KL divergence distribution as expressed in Eq. S4. The figure assumes that $\sigma_{0}^{2} = 2$ and *n* = 25 (on the left side) or *n* = 50 (on the right side). Each plot shows the central 95% of the sampling distribution for $\hat{\sigma}^{2}$. When $\sigma_{0}^{2}>\sigma_{1}^{2}$, the KL divergence probabilities are smaller than the asymptotic sampling distribution. When $\sigma_{0}^{2}<\sigma_{1}^{2}$, the KL divergence probabilities are larger than the asymptotic sampling distribution. The overall percent change of the correction reduces as the number of samples *n* grows.


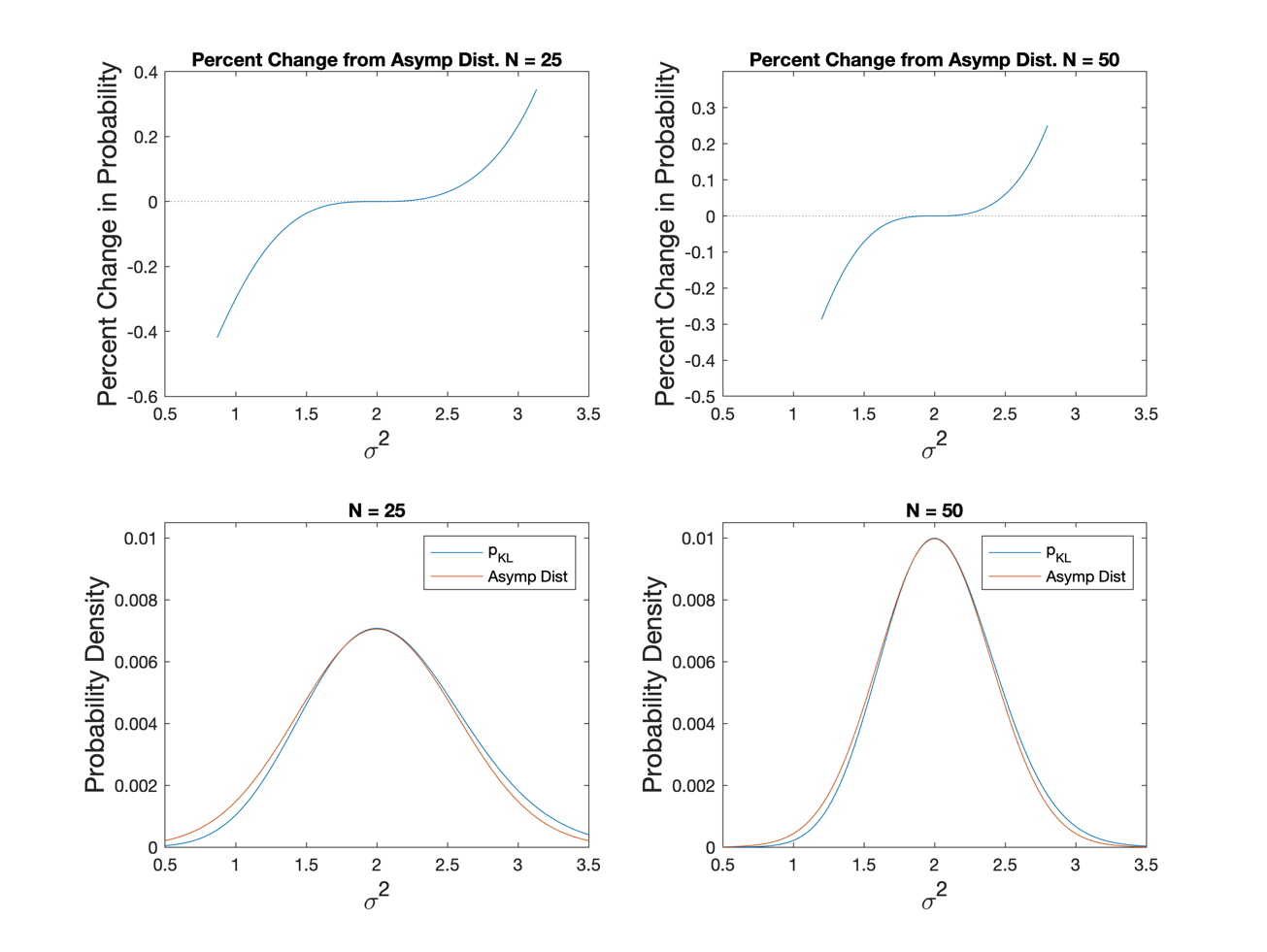


***Fig. S1.*** *The difference between the uncorrected probabilities derived from the KL divergence surface (Uncorrected* $p_{KL}$*) and the asymptotic sampling distribution for estimating the variance of a normal Gaussian distribution (Asymp. Dist.).*

**Approximate Numerical Sampling Distributions for Binomial Likelihoods**

The binomial distribution is often used in behavioral and cognitive modeling for studying
dichotomous variables such as choice and correct/incorrect response. Logistic regression provides an inferential model for dichotomous response variables with known estimation properties that is already widely used in the social sciences. However, there currently does not exist a method for understanding the estimation properties of a broader class of cognitive models applied to dichotomous response variables such as Expected Utility Theory, Cumulative Prospect Theory and the Generalized Context Model. We therefore test the applicability of our KL divergence surface to generate accurate sampling distributions for a broader class of binary prediction models. The two corrections $\alpha$ and $\beta$ defined above can be applied to the KL divergence surface to generate the exact asymptotic sampling distribution for estimating the mean and variance of a normal Gaussian distribution. This is our main result given in Eq. 2$.$ The Gaussian is a location-scale distribution in the exponential family. We are interested in extending our KL divergence distribution to the Binomial which is a one parameter distribution in the exponential family. Because the Binomial distribution can be approximated by a normal distribution when sample sizes are large enough, we test the generalizability of Eq. 2 to generate approximate sampling distributions for models with Binomial likelihoods.

The Binomial distribution has one free parameter: π is the probability of a success. This distribution is also controlled by *n,* the number of draws from the distribution, but this is defined by the experiment. The mean and variance of the binomial are both a function of π (the only parameter), so we do not need the $\alpha$ correction and it is set to 1.

The likelihood for the Binomial distribution given *n* observations of *yi* = {0, 1} is given by,

$$L\left( \pi| \boldsymbol{y},n \right)= \prod_{i=1}^{n} \pi^{y_{i}}\left[ 1-\pi\right]^{(1-y_{i})} (S15)$$

The approximate sampling distribution for the parameter $\pi$ is given by,

$$p_{KL}\left( \hat{\pi}= \pi_{1} | n \right)\propto\exp\left( -1*D_{KL}\left( L\left( \pi_{0} | \boldsymbol{y}\boldsymbol{,}n \right)||L\left( \pi_{1} | \boldsymbol{y}\boldsymbol{,}n \right) \right)-\beta\right)$$

$$=\exp\left( -n*\left[ \pi_{0}\ln\left( \frac{\pi_{0}}{\pi_{1}} \right)+\left( 1-\pi_{0} \right)\ln\left( \frac{{1-\pi}_{0}}{{1-\pi}_{1}} \right) \right]-\beta\right), (S16)$$

where

$$\beta= -n*\frac{{\pi_{1}(1-\pi}_{1})}{{\pi_{0}(1-\pi}_{0})}\ln\left( \frac{\sqrt{{\pi_{1}(1-\pi}_{1})}}{\sqrt{{\pi_{0}(1-\pi}_{0})}} \right)+ \frac{\left( {\pi_{1}(1-\pi}_{1}) \right)^{2} -({{\pi_{0}(1-\pi}_{0}))}^{2}}{\frac{\left( 2{\pi_{0}(1-\pi}_{0}) \right)^{2}}{n}}. (S17)$$

Figure S2 displays the sampling distribution computed using Eq. S16 along with an empirical simulation of the sampling distribution for $\hat{\pi}$ based on drawing a sample of *n* observations $y_{i}$ = {0,1} from a binomial distribution and computing $\hat{\pi}$ as the mean of ***y*** using 50,000 replications (denoted $p_{Emp}$). When *n* = 25 and π = 12/25, $D_{KL}(p_{Emp}||p_{KL}) = 0.0043$ and the $p_{KL}$ 95% confidence interval is identical to the empirically simulated interval [0.28, 0.64]. When *n* = 50 and π = 12/50, $D_{KL}(p_{Emp}||p_{KL}) =$0.0071 and the $p_{KL}$ 95% confidence interval is [0.14, 0.36], slightly narrower than the empirically simulated interval. While not an exact match with the empirically simulated distribution, the $p_{KL}$ distribution is a close approximation and does not require extensive simulation to compute.


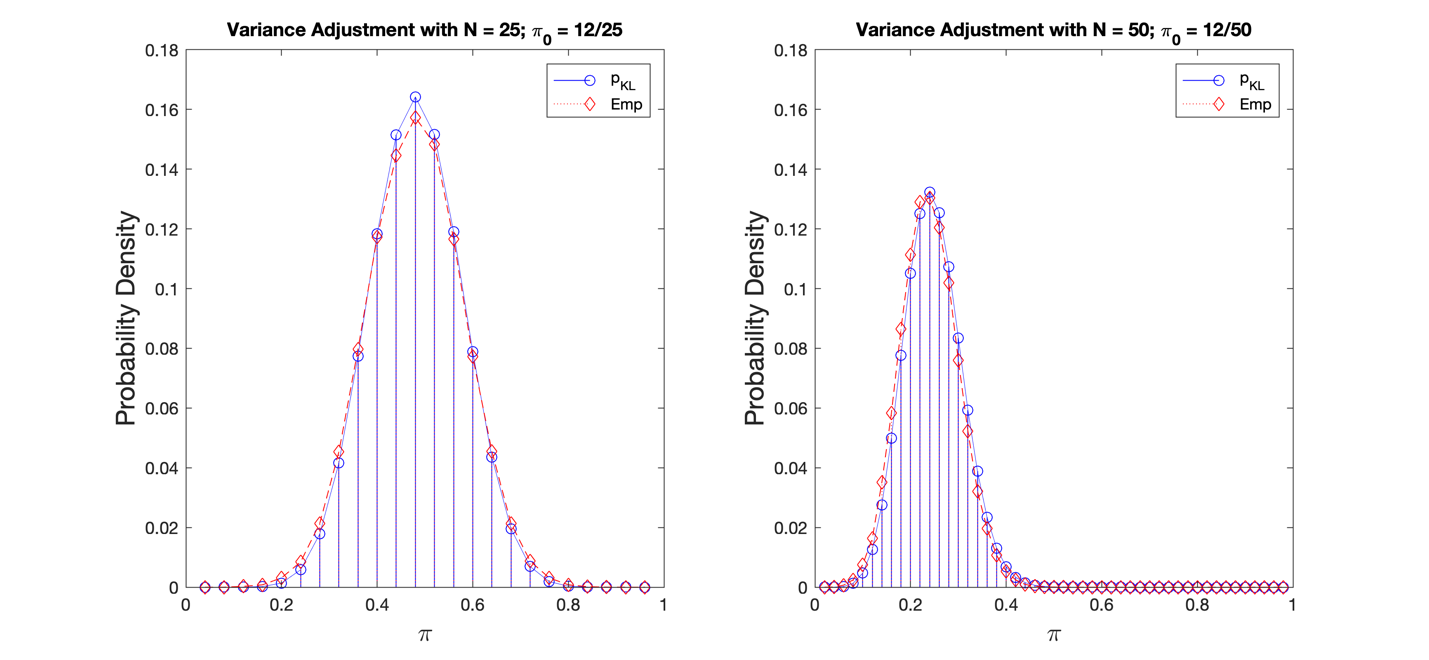


***Fig. S2.*** *Comparison of the sampling distribution for the probability of a binomial distribution (*$\hat{\pi}$*) derived from the KL divergence surface (*$p_{KL}$*) and from an empirical simulation of 50,000 estimates (*$p_{Emp}$*).*

Finally, we checked on the effect of the $\beta$ correction being generalized to the binomial case and found it is indeed improving the accuracy of the $p_{KL}$ distribution. Without the $\beta$ correction, when *n* = 25 and π = 12/25, $D_{KL}(p_{Emp}||p_{KL}) = 0.007$ and when *n* = 50 and π = 12/50, $D_{KL}(p_{Emp}||p_{KL}) =$0.02.

**Establishing the validity of the KL sampling distribution with simulation.**

For each of the KL sampling distributions displayed in the main article (Figures 4, 6, and 8), we performed a simulation study to simulate an empirical sampling distribution by using the focal parameter set to generate choices from each behavioral model, then calculate the maximum likelihood estimate of the parameters based on these choices. We replicated this process 5,000 times and used the recovered estimates to form an empirical sampling distribution (denoted $p_{Emp}$). This empirical sampling distribution is the closest ground truth that we can use to test whether the KL sampling distributions are valid representations of the true sampling distribution. Due to the computation and time requirements of this approach, we only perform these simulations for a single set of parameter values for each model.

***The Generalized Context Model.*** Figure 4 in the main manuscript displays the sampling distribution for the Generalized Context Model (GCM). To evaluate the validity of the sampling distribution, we estimated 5,000 sets of parameters by simulating choices from the GCM with parameters [$w_{0}$, $c_{0}$] = [0.5, 1.0] and generated a smoothed distribution surface from the empirical estimates (the simulation took 396 seconds to run). The KL sampling distribution very closely approximates the empirically simulated distribution (Condition 1, $D_{KL}(p_{KL}||p_{Emp}) = 0.67$; Condition 2, $D_{KL}(p_{KL}||p_{Emp}) = 0.68$; Condition 3, $D_{KL}(p_{KL}||p_{Emp}) = 0.06$; Condition 4, $D_{KL}(p_{KL}||p_{Emp}) = 0.06$;). The KL and empirical distributions are shown for each condition side-by-side in Figure S3. Due to the problematic shape of the sampling distribution, the empirical correlations are much stronger and result in a strongly bimodal distribution. This shows that the parameters in these two conditions are not uniquely identifiable as suggested by the KL sampling distribution. The hierarchical Bayesian approach employed by Barlema et al. (2014) might not have revealed this because the prior for the parameter values may have pushed the estimation back to a unimodal shape.


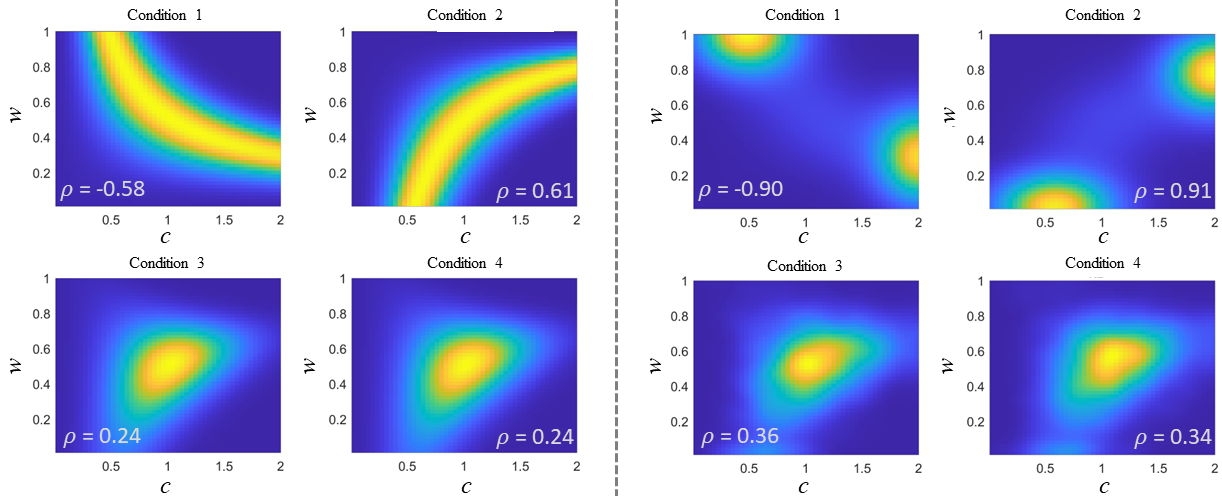
***Fig. S3.*** *Comparison of the KL sampling distribution (left) with an empirically simulated sampling distribution (right) for GCM with parameter values* $w_{0}$ *= 0.5 and c0 = 1 separately for each condition.*

***Cumulative Prospect Theory.*** Figure 6 in the main manuscript displays the sampling distribution for the null hypothesis used to test parameters for Cumulative Prospect Theory (CPT) estimated by Glockner and Pachur (2012). We performed a hypothesis test by constraining two of the model parameters to be equal to 1. These two parameters were statistically dependent on the remaining parameters that we did not constrain. To reduce the effect of these dependencies on the null hypothesis test, we adapted a procedure developed by Huang, Broomell, & Golman (2024) to identify the most conservative null hypothesis for this data. This process involves identifying the set of null parameters (with some constraints applied) that have the highest likelihood of generating the estimated parameter values.

For the cumulative prospect theory results, we leveraged the computational speed of the KL sampling distributions to generated distributions for all combinations of α in the interval [0.55, 0.9] in 15 equal increments and ϵ in the interval [0.02, 0.1] in 15 equal increments while constraining γ = λ = 1. For each constrained sampling distribution, we looked at the likelihood of the estimated parameter values [$\hat{\alpha}$, $\hat{\gamma}$, $\hat{\lambda}$, $\hat{\epsilon}$] = [0.71, 0.72, 1.35, 0.08], and retained the values of α and ϵ that maximized likelihood ($\alpha_{0}=0.75$ and $\epsilon_{0}=0.06$). This procedure took 3,956 seconds to run using the KL sampling distribution approach and would be prohibitively longer to run if each sampling distribution was empirically simulated. The likelihood search results are displayed in Table S1.

**Table S1.** The likelihood of the estimated parameters [$\hat{\boldsymbol{\alpha}}$, $\hat{\boldsymbol{\gamma}}$, $\hat{\boldsymbol{\lambda}}$, $\hat{\boldsymbol{\epsilon}}$] = [0.71, 0.72, 1.35, 0.08] for different values of $\boldsymbol{\alpha}$ and $\boldsymbol{\epsilon}$ for the constrained null hypothesis. The table displays likelihood x 10^5^ with the maximal value bolded for $\boldsymbol{\alpha}_{\boldsymbol{0}}\boldsymbol{=0.75}$ and $\boldsymbol{\epsilon}_{\boldsymbol{0}}\boldsymbol{=0.06}$. The null parameters differ from the estimated parameters because of the parameter dependencies.

|  | $\backslash epsilon$ | | | | | | | | | | | | | | |
| --- | --- | --- | --- | --- | --- | --- | --- | --- | --- | --- | --- | --- | --- | --- | --- |
| $\backslash alpha$ | **0.020** | **0.026** | **0.031** | **0.037** | **0.043** | **0.049** | **0.054** | **0.060** | **0.066** | **0.071** | **0.077** | **0.083** | **0.089** | **0.094** | **0.100** |
| **0.550** | 0.00 | 0.00 | 0.00 | 0.00 | 0.00 | 0.00 | 0.00 | 0.00 | 0.00 | 0.00 | 0.00 | 0.00 | 0.00 | 0.00 | 0.00 |
| **0.575** | 0.00 | 0.00 | 0.00 | 0.00 | 0.00 | 0.00 | 0.00 | 0.00 | 0.00 | 0.00 | 0.00 | 0.00 | 0.00 | 0.01 | 0.01 |
| **0.600** | 0.00 | 0.00 | 0.00 | 0.00 | 0.00 | 0.00 | 0.00 | 0.00 | 0.00 | 0.00 | 0.01 | 0.01 | 0.03 | 0.05 | 0.09 |
| **0.625** | 0.00 | 0.00 | 0.00 | 0.00 | 0.00 | 0.00 | 0.00 | 0.00 | 0.01 | 0.02 | 0.04 | 0.09 | 0.16 | 0.28 | 0.43 |
| **0.650** | 0.00 | 0.00 | 0.00 | 0.00 | 0.00 | 0.00 | 0.01 | 0.02 | 0.05 | 0.12 | 0.24 | 0.44 | 0.69 | 0.97 | 1.25 |
| **0.675** | 0.00 | 0.00 | 0.00 | 0.00 | 0.00 | 0.01 | 0.04 | 0.12 | 0.28 | 0.56 | 0.93 | 1.37 | 1.75 | 1.88 | 1.81 |
| **0.700** | 0.00 | 0.00 | 0.00 | 0.00 | 0.02 | 0.08 | 0.24 | 0.57 | 1.08 | 1.69 | 2.17 | 2.23 | 1.96 | 1.42 | 0.67 |
| **0.725** | 0.00 | 0.00 | 0.00 | 0.03 | 0.13 | 0.42 | 0.97 | 1.75 | 2.40 | 2.44 | 1.87 | 0.96 | 0.26 | 0.02 | 0.00 |
| **0.750** | 0.00 | 0.00 | 0.03 | 0.17 | 0.59 | 1.41 | 2.30 | **2.50** | 1.83 | 0.67 | 0.06 | 0.00 | 0.00 | 0.00 | 0.00 |
| **0.775** | 0.00 | 0.02 | 0.16 | 0.70 | 1.70 | 2.40 | 1.91 | 0.59 | 0.02 | 0.00 | 0.00 | 0.00 | 0.00 | 0.00 | 0.00 |
| **0.800** | 0.01 | 0.11 | 0.66 | 1.73 | 2.04 | 0.87 | 0.03 | 0.00 | 0.00 | 0.00 | 0.00 | 0.00 | 0.00 | 0.00 | 0.00 |
| **0.825** | 0.04 | 0.47 | 1.51 | 1.54 | 0.17 | 0.00 | 0.00 | 0.00 | 0.00 | 0.00 | 0.00 | 0.00 | 0.00 | 0.00 | 0.00 |
| **0.850** | 0.21 | 1.12 | 1.10 | 0.01 | 0.00 | 0.00 | 0.00 | 0.00 | 0.00 | 0.00 | 0.00 | 0.00 | 0.00 | 0.00 | 0.00 |
| **0.875** | 0.62 | 0.91 | 0.00 | 0.00 | 0.00 | 0.00 | 0.00 | 0.00 | 0.00 | 0.00 | 0.00 | 0.00 | 0.00 | 0.00 | 0.00 |
| **0.900** | 0.77 | 0.00 | 0.00 | 0.00 | 0.00 | 0.00 | 0.00 | 0.00 | 0.00 | 0.00 | 0.00 | 0.00 | 0.00 | 0.00 | 0.00 |

To evaluate the validity of the sampling distribution, we estimated 5,000 sets of parameters by simulating choices from CPT with the null parameters [$\alpha_{0}$, $\gamma_{0}$, $\lambda_{0}$, $\epsilon_{0}$] = [0.75, 1.00, 1.00, 0.06], and generated a smoothed distribution surface from the empirical estimates (the simulated estimates took 379 seconds and the smoothed empirical distribution took 1,124 seconds). The KL sampling distribution very closely approximates the empirically simulated distribution ($D_{KL}(p_{KL}||p_{Emp}) = 1.38$). The CPT bivariate marginal plots for the KL and empirical distributions are shown side-by-side in Figure S4. The empirical distribution is less smooth than the CRRA model, which is due to the higher parameter dimensionality, increased model complexity, and reduced sample size relative to the CRRA analysis.


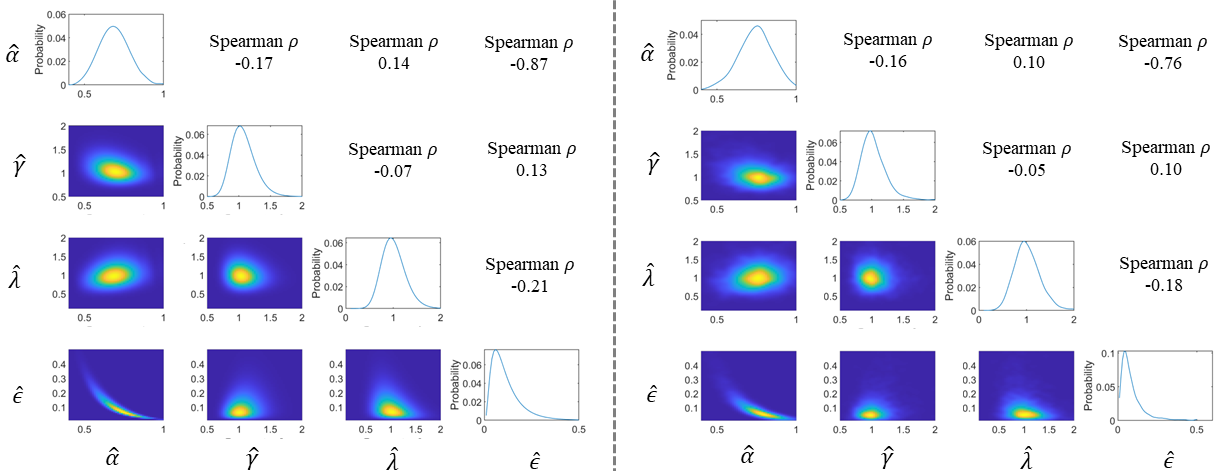
***Fig. S4.*** *Comparison of the KL sampling distribution (left) with an empirically simulated sampling distribution (right) for CPT with parameter values* $\alpha_{0}$*= 0.75,* $\gamma_{0}$ *= 1.00,* $\lambda_{0}$*= 1.00, and* $\epsilon_{0}$ *= 0.06.*
